# Immunotherapy for rapid bone marrow conditioning and leukemia depletion that allows efficient hematopoietic stem cell transplantation

**DOI:** 10.1136/jitc-2025-011888

**Published:** 2025-06-27

**Authors:** Giada Dal Collo, Srdjan Grusanovic, Milad Rasouli, Antoinette van Hoven-Beijen, Yvonne M Mueller, Zara-Li van der Sande, Martin van Hagen, Jan J Cornelissen, Yun He, Yuandong Wang, Emma De Pater, Vincent H J van der Velden, Marc H G P Raaijmakers, Meritxell Alberich-Jorda, Jiuqiao Zhao, Peter D Katsikis, Stefan J Erkeland

**Affiliations:** 1 Department of Immunology, Erasmus MC University Medical Center Rotterdam, Rotterdam, The Netherlands; 2 Czech Academy of Sciences, Prague, Czech Republic; 3 Department of Hematology, Erasmus MC Universitair Medisch Centrum Rotterdam, Rotterdam, The Netherlands; 4 Harbour Biomed Shanghai Co Ltd, Shanghai, Shanghai, China; 5 Department of Pediatric Haematology and Oncology, Harbour Biomed Shanghai Co Ltd, Shanghai, Shanghai, China; 6 Department of Pediatric Haematology and Oncology, Motol University Hospital Department of Pediatrics, Prague, Czech Republic

**Keywords:** Immunotherapy, Bispecific T cell engager - BiTE, Leukemia, Pharmacokinetics - PK, Stem cell

## Abstract

Hematopoietic stem cell transplantation (HSCT) is a life-saving procedure to treat hematopoietic disorders. Current bone marrow conditioning protocols create space for healthy donor stem cells by employing irradiation and/or chemotherapy, but carry severe toxicities, resulting in significant morbidity, mortality and substantial long-term complications. To develop a low-toxicity solution, we generated a bi-specific T-cell engager (BTCE) that targets CD117, an abundantly expressed receptor on hematopoietic stem and progenitor cells (HSPC) and leukemia-initiating cells (LICs). We show that the CD117×CD3 BTCE efficiently depletes in vitro and in vivo HSPCs and LICs. The CD117×CD3 BTCE was not toxic and facilitates highly efficient engraftment of human allogenic donor CD34+cells in humanized mice, thereby restoring hematopoiesis in vivo in both normal and leukemia-bearing humanized mice. We demonstrate here that a potent CD117×CD3 BTCE enables rapid HSCT in both benign and malignant conditions.

**WHAT IS ALREADY KNOWN ON THIS TOPIC**

- Current bone marrow (BM) conditioning protocols required for hematopoietic stem cell transplantation (HSCT), use irradiation and/or chemotherapy, and carry severe toxicities resulting in significant morbidity, mortality and substantial long-term complications.

**WHAT THIS STUDY ADDS**

- Here, we describe a highly specific and effective bi-specific T-cell engager (BTCE) targeting CD117, a membrane marker, that is expressed on normal and malignant hematopoietic precursors. We show that our CD117×CD3 BTCE has a very short half-life and efficiently depletes human hematopoietic stem cells and leukemia-initiating cells in preclinical humanized mouse and patient-derived xenograft models. CD117×CD3 BTCE-mediated BM conditioning allows robust allogenic HSCT in humanized mice without obvious toxicities.

**HOW THIS STUDY MIGHT AFFECT RESEARCH, PRACTICE OR POLICY**

- Our novel BTCE approach holds promise for safe and efficient BM conditioning prior to HSCT in both benign and malignant conditions.

## Introduction

 Hematopoietic stem cell transplantation (HSCT) has emerged as an effective therapeutic approach to treat benign and malignant hematopoietic diseases, including myeloid and lymphoid malignancies, inherited metabolic disorders, hemoglobinopathies, bone marrow failure (BMF)—and patients with inborn errors of immunity.^1^,^6^ The current bone marrow (BM)-conditioning for HSCT typically includes chemotherapy and/or total body irradiation to create space in the BM for the engraftment of donor hematopoietic stem and progenitor cells (HSPCs). However, this approach is hampered by associated short and long-term toxicities, limiting its application for the majority of patients with leukemia who are not clinically fit enough.^7^,^11^ This high toxicity also hinders the rapidly developing gene transfer or gene editing strategies to correct inborn errors of immunity, metabolic diseases, hemoglobinopathies, and BMF, as HSCT is a prerequisite for such therapies.^12^ Therefore, improved BM-conditioning protocols prior to HSCT are needed to minimize toxicities. CD117 (also known as cKIT), a receptor tyrosine kinase abundantly expressed on HSPCs and leukemia-initiating cells (LICs), is critical for cellular survival and proliferation, and can serve as a target for HSPC and LIC elimination and BM-conditioning.^13^

Previously, a monoclonal IgG antibody that blocks mouse CD117 (anti-cKIT, clone ACK2) was developed to rapidly deplete HSPCs in mice and demonstrated that blocking CD117 function with ACK2 anti-CD117 antibody leads to rapid depletion of HSPCs from the BM niches, thereby facilitating long-term engraftment of donor hematopoietic stem cells (HSCs).^14^ However, it has been demonstrated that targeting CD117 with a CD117‐blocking antibody may not be sufficient to fully eliminate all LICs and to achieve high donor cell chimerism after HSCT.^15 16^ Therefore, novel anti-CD117 antibody-drug conjugates,^17 18^ anti-CD117 chimeric antigen receptor cells-T (CAR-T),^19^,^21^ a CD117-targeting bispecific antibody^22^ and CD117 epitope-edited donor HSCs in combination with anti-CD117 CAR-T cells have been explored for improved targeting of acute myeloid leukemia (AML).^23^ Hence, the most studied anti-CD117 reagent is briquilimab (JSP191, formerly AMG191), an anti-human CD117 antibody validated in preclinical mouse xenograft models and immunocompetent cynomolgus macaques.^24^

Briquilimab is tested as a conditioning agent in patients with severe combined immunodeficiencies (SCID, NCT02963064), myelodysplastic syndrome (MDS)/AML (NCT04429191), sickle cell disease (NCT05357482) and Fanconi anemia (FA, NCT04784052) undergoing allogenic HSCT. Although the initial results in humans are promising with no detectable infusion-related toxicities, briquilimab has a long half-life in the body, allowing HSCT at the earliest 2–3 weeks post-treatment, the time needed for antibody plasma levels to decline to 500–2,000 ng/mL, a concentration that allows donor HSPCs engraftment in the BM.^24^,^26^ However, such delayed HSCT results in poor donor cell chimerisms after HSCT due to recovery of the host HSC population in the BM of the host.^27^

Because of these limitations, a more efficient approach for BM-conditioning may require reduced half-life and direct killing of HSPCs that allow rapid HSCT after treatment. Bi-specific T-cell engagers (BTCE) consist of two single-chain variable fragments (scFvs) from different antibodies, of which one scFv binds to the CD3 receptor of T cells and the other to a specific target cell-associated epitope. Such BTCE can facilitate the interaction of T cells with target cells, directly killing those targets.^28^

Here, we generated and evaluated a novel CD117×CD3 BTCE as a rapid strategy for efficient BM-conditioning and LIC depletion prior to HSCT. We report here that CD117×CD3 BTCE with a very short half-life can safely and potently deplete normal and malignant HSPCs in preclinical mouse models, enabling high levels of donor HSC engraftment and chimerism. These findings indicate the high potential of BTCE for clinical applications of safe and efficient HSCT.

## Material and methods

See online supplemental material and methods.

## Results

### CD117×CD3 BTCE binds specifically to human CD117 and CD3 positive cells

**Figure 1:**
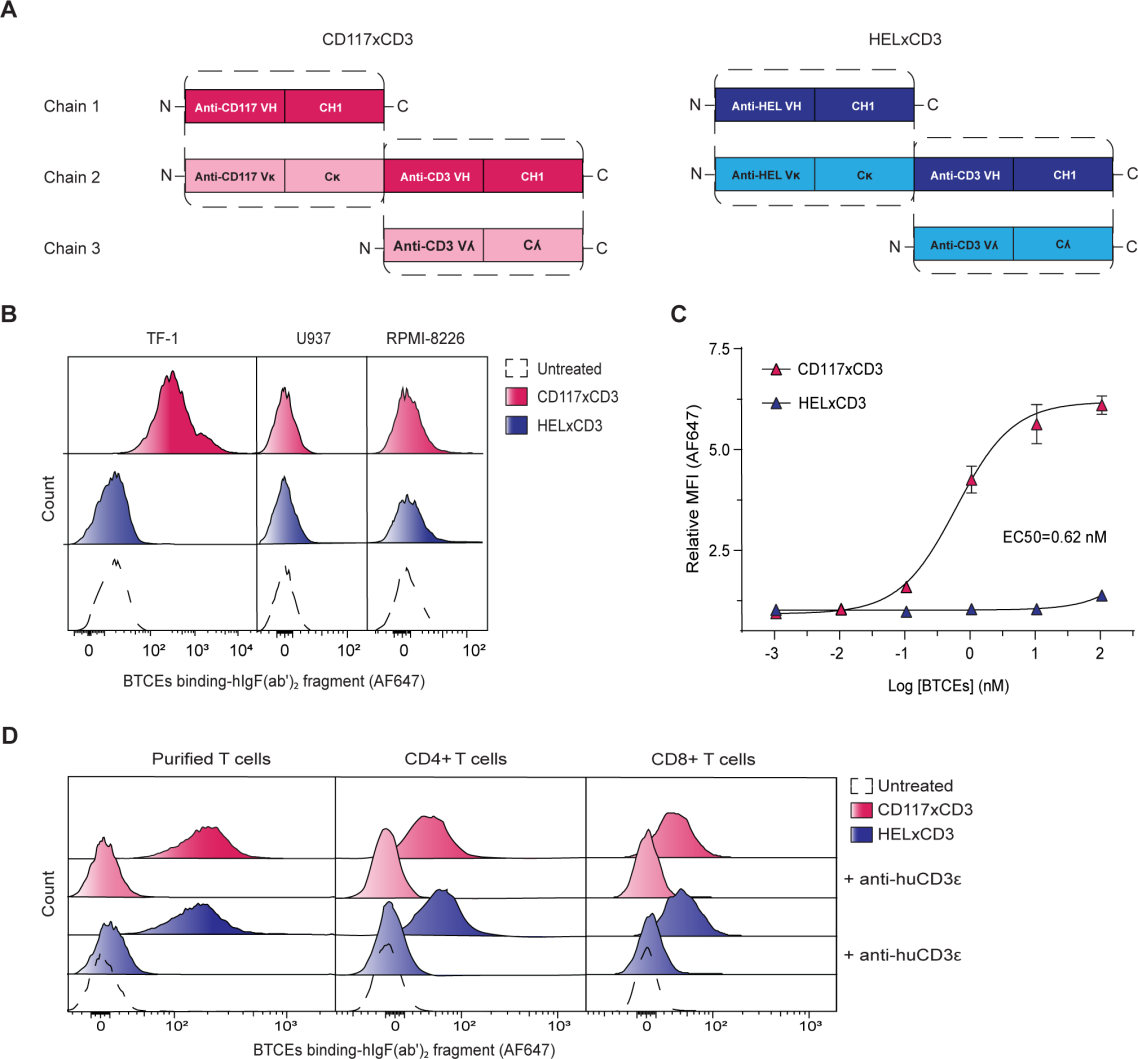
Binding specificity of CD117×CD3 BTCE. (**A**) Schematic representation of CD117×CD3 and HEL×CD3 BTCEs. Heavy chains (CH and VH) are in dark colors, and light chains (Cκ, Cλ, Vκ and Vλ) are in light colors. (**B**) FACS plots showing binding of CD117×CD3 and control HEL×CD3 BTCEs to CD117 receptors on TF-1 cells. Negative controls U937 and RPMI-8226 do not express CD117 or CD3. Data are representative of three independent experiments. (**C**) EC50 of CD117×CD3 BTCE binding to TF-1 cells as determined by flow cytometry. Data are plotted as relative median fluorescent intensity and fitted to a dose-response non-linear regression model. Data are representative of three independent experiments. Error bars indicate SEM. (**D**) FACS plots showing the binding of CD117×CD3 and control HEL×CD3 BTCEs to CD3 receptor expressed on purified human T cells, CD4+ and CD8+ T-cell subsets in human PBMCs. PBMCs and purified T cells pretreated with blocking anti-CD3ε antibodies are also shown. Data are representative of three independent experiments. BTCE, bi-specific T-cell engager; FACS, fluorescent-activated cell sorter; PBMC, peripheral blood mononuclear cell.

A schematic representation of CD117×CD3 and HEL×CD3 BTCEs (HEL: hen egg lysozyme, a negative control) is shown in figure 1A. For CD117×CD3 BTCE, the VL-VH part of the hSR1 clone^29^ and SP34-2 clone^30^ antibodies was used, respectively. The complete amino acid sequences of our reagents are given in online supplemental figure 1. BTCE reagents consisted of a single band in SDS-PAGE (Sodium Dodecyl Sulfate Polyacrylamide Gel Electrophoresis), SEC-HPLC (Size Exclusion-High Performance Liquid Chromatography) and mass spectrometric analysis and revealed a mass of 94.5 KDa and 93.7 KDa for CD117×CD3 BTCE and HEL×CD3 BTCE, respectively, with established purities of approximately 95% (online supplemental figure 2).

We first investigated the binding specificity of CD117×CD3 BTCE and found that in vitro CD117×CD3 BTCE binds to TF-1 cells, an AML cell line that expresses the CD117 receptor and lacks CD3 expression, but not to RPMI-8226 and U937 cells, two hematopoietic cell lines that do not express either CD117 or CD3 (figure 1B). The HEL×CD3 control BTCE does not bind to TF-1 cells (figure 1B). CD117×CD3 BTCE binds to TF-1 cells in a dose-dependent manner with a half maximal effective concentration of 0.62 nM (figure 1C). We next tested in vitro the binding capacity of CD117×CD3 and HEL×CD3 BTCEs to the CD3 receptor, using human peripheral blood mononuclear cells (PBMCs) and purified human T cells. We found that both CD117×CD3 and HEL×CD3 BTCEs bind specifically via CD3 to purified T cells, CD4+ and CD8+ T-cell subsets in PBMCs (figure 1D). This binding was specific as it could be blocked by anti-CD3 monoclonal antibodies targeting the same epitope (figure 1D). In addition, CD117×CD3 and HEL×CD3 BTCEs do not bind to cells without CD3 expression, such as U937 and RPMI-8226 cells (figure 1B). Together, these data show that CD117×CD3 BTCE binds specifically to CD117 and CD3.

### CD117×CD3 BTCE triggers potent T cell-mediated cytolysis of human CD117+ cells

**Figure 2:**
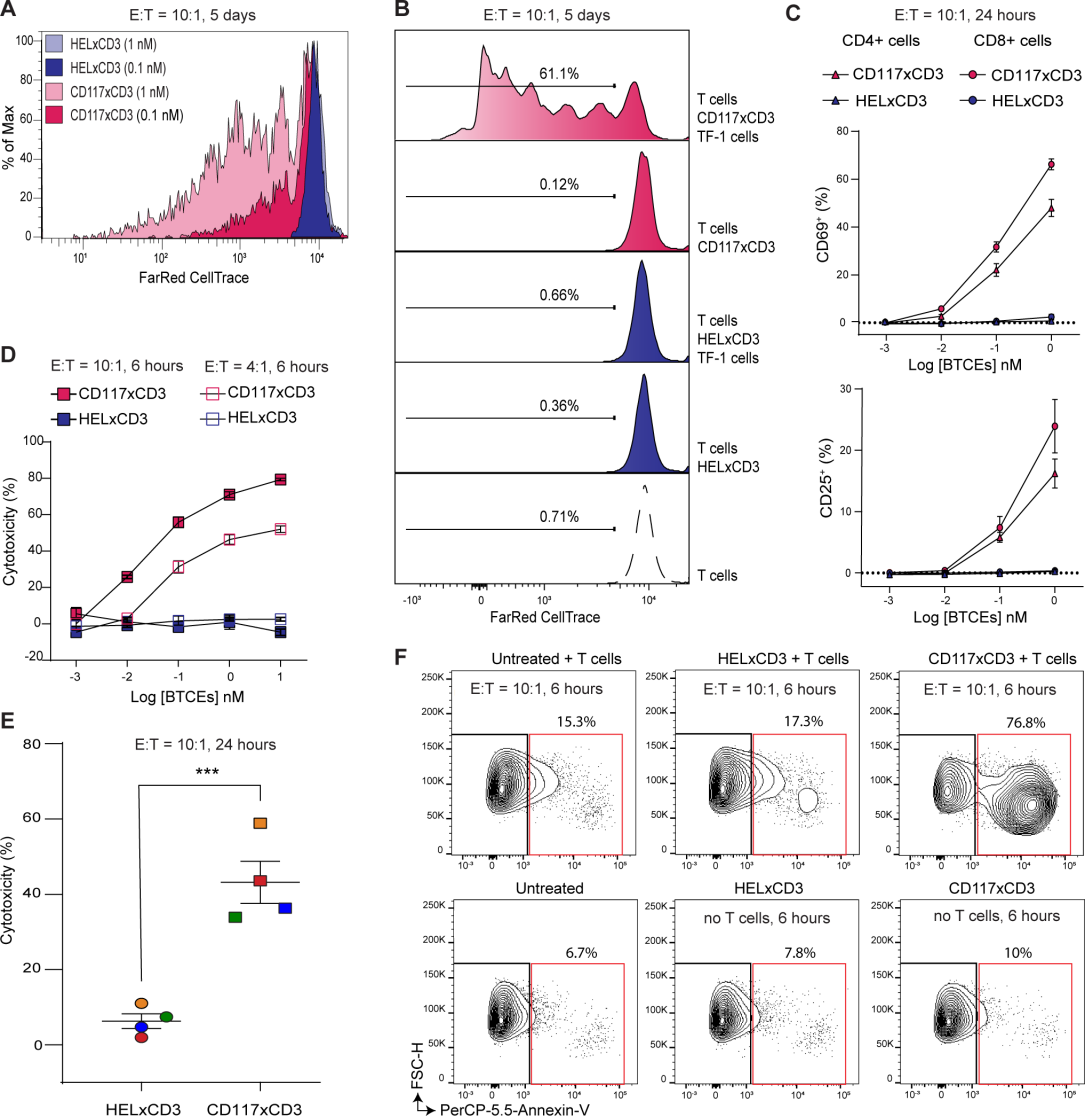
Cytotoxic activity of CD117×CD3 BTCE in vitro. (**A**) Representative FACS plot showing CD117×CD3 BTCE dose-dependent T-cell proliferation, as determined by FarRed CellTrace signal dilution, when human T cells and TF-1 target cells are co-cultured. Data are representative of three independent experiments. (**B**) Representative FACS plots showing the percentage of T cells with diluted FarRed CellTrace signals in the indicated conditions. Data are representative of three independent experiments. (**C**) Percentage of CD4+ (triangles) and CD8+ (circles) T-cell subsets expressing the indicated activation markers CD69 (top) and CD25 (bottom) in the presence of TF-1 target cells and indicated reagents are shown. Data points are the average of three experiments. (**D**) Dose-dependent CD117×CD3 BTCE-mediated cytotoxicity of TF-1 cells after 6 hours of co-culture with human T cells at indicated E:T ratios. E:T ratio is the ratio of T cells to TF-1 cells. (**E**) Percentage of T cell-mediated cytotoxicity of primary human BM CD34+cells (n=4 donors) when treated with 1.1 nM of indicated BTCE and co-culture with non-HLA-matched healthy donor-derived T cells (E:T ratio of 10:1) for 24 hours is given. Colors indicate individual BM CD34+cell samples. The two-tailed Student’s t-test was used for statistical analysis. ***p<0.001. (**F**) FACS plots showing Annexin-V+TF-1 cells (red boxes) after 6 hours of co-culture with T cells (upper panels, E:T ratio of 10:1) or without T cells (lower panels) and indicated BTCE treatments. Error bars in C, D and E represent SEM. BM, bone marrow; BTCE, bi-specific T-cell engagers; E:T, effector to target cell ratio; FACS, fluorescent-activated cell sorter.

CD117×CD3 BTCE should not activate human T cells in the absence of their target. We therefore asked whether CD117×CD3 BTCE can simultaneously bind to human T cells and CD117+target cells and only then activate T cells. CD117×CD3 BTCE, but not HEL×CD3 BTCE, stimulated T-cell proliferation in a dose-dependent manner and only in the presence of TF-1 target cells (figure 2A,B). In accordance with this, CD117×CD3 BTCE, but not HEL×CD3 BTCE, can activate T cells in co-cultures with TF-1 target cells (figure 2C) and induce cytokines (online supplemental figure 3A). To investigate CD117×CD3 BTCE cytotoxicity against CD117+cells, we performed an in vitro T cell-dependent cellular cytotoxicity (TDCC) assay. CD117×CD3 BTCE, but not HEL×CD3 BTCE, induced TDCC in a dose-dependent manner, with up to 80% TF-1 target cell death in 6 hours (figure 2D). In addition, CD117×CD3 BTCE caused up to 60% cell death of primary human BM CD34+cells within 24 hours, when co-cultured at an effector to target cell (E:T) ratio of 10:1 with non-HLA—matched healthy donor derived T cells (figure 2E). Notably, TF-1 cells were only depleted in the presence of human T cells, indicating that the observed CD117×CD3 BTCE-mediated killing is T cell-dependent (figure 2F). Control HEL×CD3 BTCE did not show cytotoxic activity (figure 2D–F). CD117×CD3 BTCE showed no cross-reactivity to mouse T cells and no TF-1 cell killing was observed in mouse T-cell and TF-1 co-cultures, confirming the specificity of the reagent to human CD3 (online supplemental figure 3B). All together, these results demonstrate that CD117×CD3 BTCE binds specifically to human CD117-expressing target cells and human T cells and efficiently kills CD117+target cells in vitro.

### CD117×CD3 BTCE in vivo has a very short serum half-life

**Figure 3:**
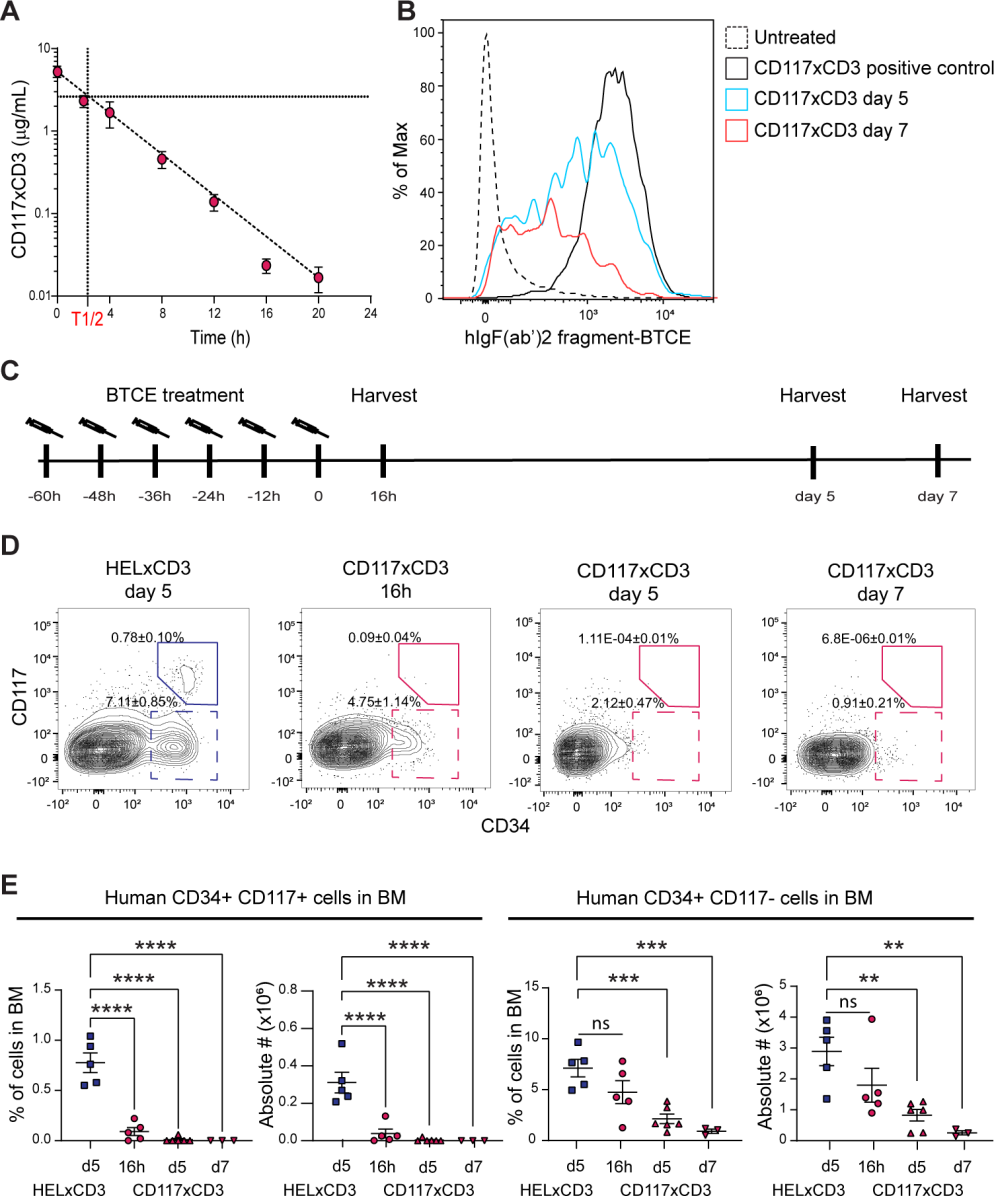
In vivo depletion of human CD117+hematopoietic stem and progenitor cells by CD117×CD3 BTCE. (**A**) Concentrations of CD117×CD3 BTCE in the serum of C57BL/6 mice at indicated time points post CD117×CD3 BTCE treatment are shown (n =3–5 animals per group). The calculated T_1/2_ is depicted in red. (**B**) FACS plot showing the level of CD117×CD3 BTCEs on human T cells in the spleen of huCD34-NSG mice at indicated time points post treatment. CD117×CD3 positive control indicates ex vivo staining with the CD117×CD3 reagent. (**C**) Schematic overview of CD117×CD3 BTCE treatment of huCD34-NSG mice and the time points of harvesting hematopoietic tissues. (**D**) Representative FACS plots showing the percentage of human CD34+CD117+ cells (closed line gate) and CD34+CD117 cells (dashed line gate) of total human CD45+cells in the BM of huCD34-NSG mice after indicated treatment and at specified time points. (**E**) Percentages and absolute numbers of CD34+CD117+ (left panel) and CD34+CD117 cells (right panel) in the BM of huCD34-NSG mice after indicated treatment and at specified time points are plotted. For figure E, a one-way analysis of variance with Dunnett’s multiple comparisons test was used for statistical analysis. Horizontal lines in E represent average. Error bars in A and E represent SEM. **p<0.01, ***p<0.001, ****p<0.0001. FDR, False Discovery Rate; BM, bone marrow; BTCE, bi-specific T-cell engagers; FACS, fluorescent-activated cell sorter; ns, not significant.

A desired property of a successful HSC depletion strategy is the rapid formation of an empty BM niche while allowing donor cells to replenish it. For this, a rapidly acting and clearing modality would be required. To determine the in vivo pharmacokinetics of CD117×CD3 BTCE, C57BL6/J mice were intraperitoneally (IP) injected with a single dose of CD117×CD3 BTCE (25 µg, 1.0 mg/kg) and blood samples from the tail vein were collected at different time points. Serum concentrations indicated that CD117×CD3 BTCE has a half-life (T½-value) of 2 hours and 19 min (figure 3A). Notably, CD117×CD3 BTCE is below the detection limit of 0.039 ng/mL after 24 hours. Clearance from the soluble fraction of blood does not exclude CD117×CD3 BTCE, which is retained in the cellular compartment, especially as CD117×CD3 BTCE binds to CD3 on human T cells. To address this, we investigated the presence of CD117×CD3 BTCE on human T cells in the spleen of CD117×CD3 BTCE-treated immunodeficient NSG mice reconstituted with human CD34+cells (huCD34-NSG mice). We found that approximately 70% and 40% of human T cells were still coated with CD117×CD3 BTCE at 5 days and 7 days post-CD117×CD3 BTCE treatment, respectively, despite the rapid clearance of the reagent in the serum (figure 3B). Taken together, this demonstrates that CD117×CD3 BTCE has a short serum half-life in vivo, but may be retained longer on coated human T cells.

### Highly efficient in vivo depletion of CD117+ HSPCs by CD117×CD3 BTCE

To determine the in vivo ability of CD117×CD3 BTCE to deplete human CD34+CD117+ HSPCs, we treated huCD34-NSG mice with CD117×CD3 BTCE. To this end, huCD34-NSG mice were injected with CD117xCD3 BTCE (25 µg/injection, 1 mg/kg) six times every 12 hours (figure 3C). CD117×CD3-treated huCD34-NSG mice exhibited greatly reduced frequency and absolute number of human CD34+CD117+ BM cells compared with controls, 16 hours post-treatment (figure 3D,E). This progressed to an almost full depletion of human CD34+CD117+ HSPCs in the BM of CD117×CD3 BTCE-treated mice at 5 and 7 days post-treatment (figure 3D,E). Because of the near complete (>99%) human CD34+CD117+ HSPC depletion (figure 3D,E), we expected a decreased number of CD34+CD117– HSPCs in the BM, which are derived from CD34+CD117+ cells, at later time points. Indeed, we noted a reduction of CD34+CD117 cells at 16 hours (33%), 5 days (70%) and an almost full depletion of CD34+CD117 cells at 7 days post-treatment (figure 3D,E). The reduced number of progenitors may affect the myeloid cell population. As expected, we found decreased numbers of huCD45+CD13+ CD33+ myeloid cells in the peripheral blood (PB), BM and spleen at 5 days post-treatment (online supplemental figure 4A). Also, the total number of NK cells (huCD45+CD56+) was significantly reduced in the spleen, but not in the BM or PB, compared with HEL×CD3 BTCE-treated mice at 5 days post-treatment (online supplemental figure 4B). Among the remaining human cells, the frequency and the number of B cells (huCD45+CD19+) appeared marginally increased in the PB and spleen after CD117×CD3 BTCE treatment, respectively (online supplemental figure 4C). As expected, no depletion of mouse CD45+cells in the BM was detected in CD117×CD3 BTCE-treated huCD34-NSG mice (online supplemental figure 4D).

**Figure 4:**
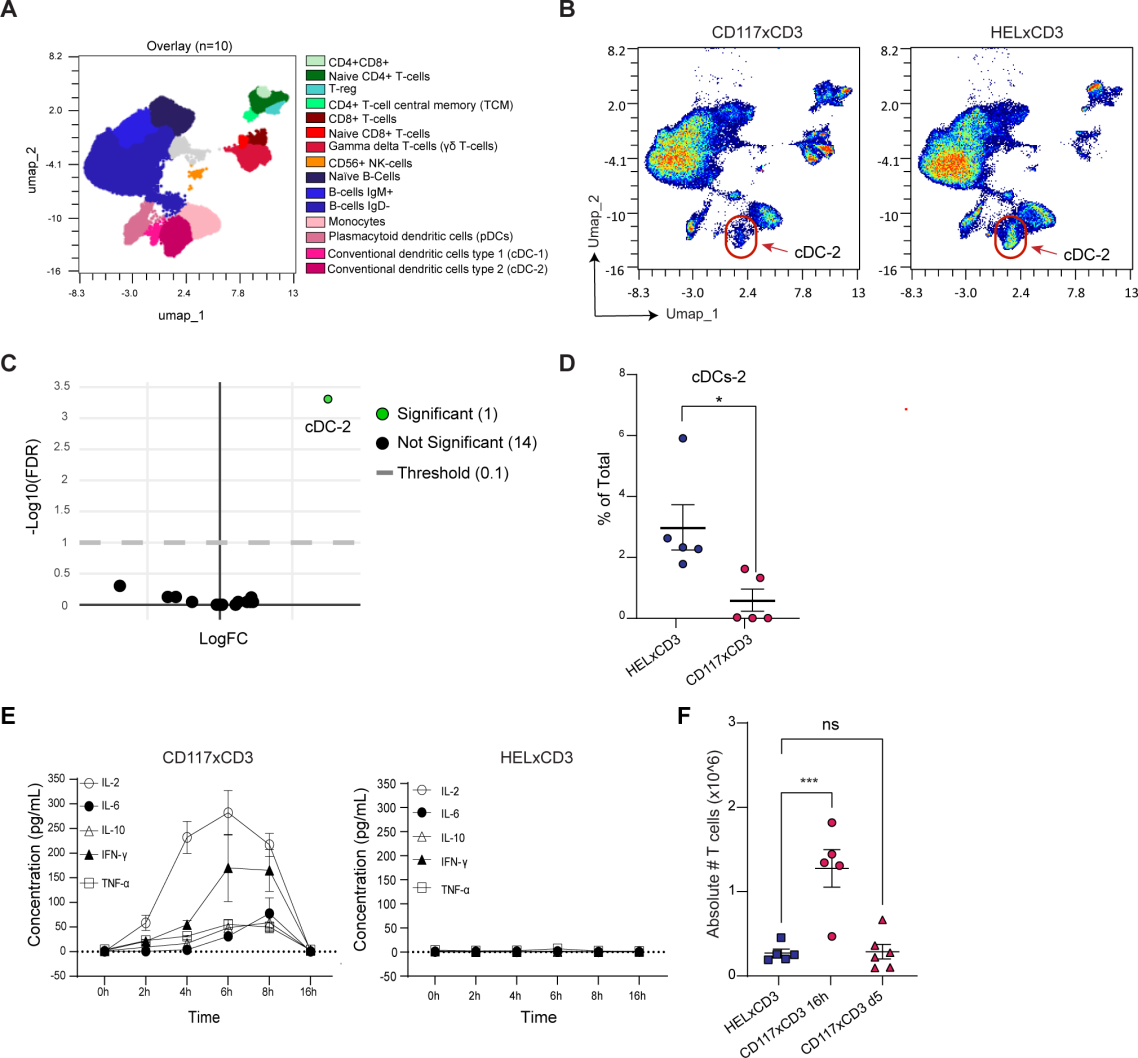
In-depth immunophenotype of the major hematopoietic cell subsets in the BM of huCD34-NSG mice after CD117×CD3 BTCE treatment. (**A**) Population cluster identification in high-dimensional 41-color flow cytometry data using Uniform Manifold Approximation and Projection (UMAP) dimensionality reduction. Cell populations in the BM of five CD117×CD3 BTCE and five HEL×CD3 BTCE-treated huCD34-NSG mice at 5 days post-treatment are shown in an overlay plot. (**B**) Overlay UMAP plots of BM cells of five representative huCD34-NSG mice treated with indicated BTCE and isolated at 5 days post-treatment are shown. cDC-2 are indicated by red circles. (**C**) Volcano plot showing the fold change of five individual BM cell populations of CD117×CD3 BTCE-treated huCD34-NSG compared with HEL×CD3-treated controls. A significant positive fold change indicates that the cellular population was decreased in CD117×CD3 BTCE-treated huCD34-NSG mice. (**D**) Percentage of cDCs-2 in the BM of huCD34-NSG mice treated with indicated BTCE (n=5 per group) at 5 days post-treatment. The two-tailed Student’s t-test was used for statistical analysis. Lines represent average. *p<0.05. (**E**) Concentrations of indicated cytokines in the serum of CD117xCD3 BTCE-treated and HEL×CD3 BTCE-treated huCD34-NSG mice at specified time points are shown. Results are representative of two independent experiments. (**F**) Absolute numbers of human T cells in the BM of huCD34-NSG mice (n=5 or 6 animals per group) after indicated treatment and at the specified time points are shown. A one-way analysis of variance with Dunnett’s multiple comparisons test was used for statistical analysis. ***p<0.001, ns=not significant. Horizontal lines represent average. Error bars in D, E and F represent SEM. BM, bone marrow; BTCE, bi-specific T-cell engagers; cDC2, conventional dendritic cell type 2; IFN, interferon; IL, interleukin; TNF, tumor necrosis factor.

We next examined whether immune cell subsets in the BM are affected by CD117×CD3 BTCE treatment in vivo. We applied the 41-color spectral flow cytometry to our BM samples of BTCE-treated huCD34-NSG mice at 5 days post-treatment, to in-depth immunophenotype the major immune cell subsets (figure 4A). CD117×CD3 BTCE treated humanized mice exhibited no significant difference in the frequency and activation of immune cell populations, apart from a significant reduction in the number of conventional dendritic cell type 2 population (cDC-2) (figure 4B–D), which is known to contain a subpopulation that expresses CD117.^31^

Further supporting the T cell-mediated activity of CD117×CD3 BTCE in treated humanized mice was the finding of transient secretion of T cell-specific cytokines interleukin (IL)-2 and interferon-γ, and to a much lesser extent other cytokines such as IL-6, IL-10 and tumor necrosis factor-α (figure 4E). The peak of inflammatory cytokines induction was 6–8 hours and levels were back to normal 16 hours post-treatment, suggesting a rapid and transient activation of human T cells (figure 4E). Notably, HEL×CD3 BTCE did not induce any cytokines (figure 4E). CD117×CD3 BTCE treatment was accompanied by a transient increase of T-cell numbers in the BM at 16 hours post-treatment, which returned to normal levels by 5 days post-treatment, suggesting a transient human T-cell recruitment in the BM (figure 4F).

Together, these data demonstrate a specific and efficient in vivo depletion of HSPCs by CD117×CD3 BTCE that also affects mainly the development of the myeloid cells and cDC-2 population without the induction of a cytokine storm.

### Highly efficient allogenic HSCT after CD117×CD3 BTCE-mediated BM-conditioning

**Figure 5:**
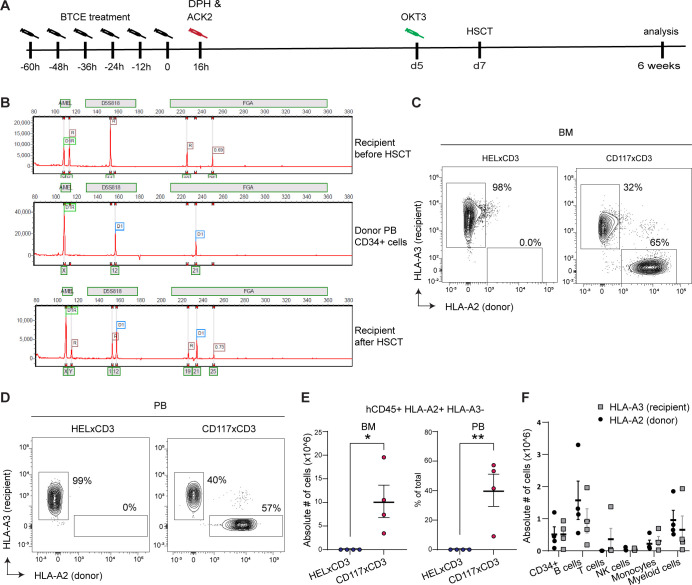
CD117×CD3 BTCE treatment allows rapid and highly efficient allogenic HSCT in humanized NSG mice. (**A**) Schematic overview of the treatments for CD117×CD3 BTCE-mediated HSPC depletion and allogenic HSCT of huCD34-NSG mice. Control mice received HEL×CD3 BTCE. ACK2 anti-mouse CD117 blocking antibody. (**B**) STR genotyping of DNA isolated from human BM cells at 6 weeks post transplantation of huCD34-NSG mice. Recipient BM cells (top), CD34+donor cells (middle) and BM cells from transplanted mice (bottom) are shown. Peaks indicate amplicons, and the chromosomal localization of the amplicons is indicated by the green boxes at the x-axis. (**C, D**) Representative FACS plots showing the percentage of huCD45+HLA-A2+ donor and huCD45+HLA-A3+ recipient cells in the BM and PB of BTCE-treated huCD34-NSG mice at 6 weeks post-HSCT. FACS plots are representative of four mice per group. (**E**) Absolute numbers of human CD45+HLA-A2+ in BM (left) and percentage of CD45+HLA-A2+ cells in PB (right) in huCD34-NSG mice (n=4) treated with indicated BTCE at 6 weeks post-HSCT are shown. The two-tailed Student’s t-test was used for statistical analysis. Lines represent average. Error bars in E and F represent SEM. *p<0.05, **p<0.01. (**F**) Absolute numbers of huCD45+HLA-A2+ and huCD45+HLA-A3+ in immune cell populations in the BM of huCD34-NSG mice (n=4) treated with indicated BTCE at 6 weeks post-HSCT are shown. BM, bone marrow; BTCE, bi-specific T-cell engagers; DPH, diphenhydramine hydrochloride; HSCT, hematopoietic stem cell transplantation; PB, peripheral blood.

To test whether CD117×CD3 BTCE-mediated human CD117+HSPC depletion allows allogenic HSCT, we treated huCD34-NSG (HLA-A3+HLA-A2−) mice with six injections of CD117×CD3 BTCE or control HEL×CD3 BTCE (figure 5A). To prevent endogenous murine HSCs from taking over the empty BM niches, mice were treated with anti-mouse CD117 blocking monoclonal antibody ACK2, an antibody that does not cross-react with human CD117, 1 day after BTCE treatments (figure 5A). To avoid allogenic donor cell rejection by host human T cells and possible CD117×CD3 BTCE-coated T cells, huCD34-NSG mice were injected with anti-CD3 (OKT3) antibodies at day 5 post-treatment to delete human T cells (figure 5A).

HLA-A3+HLA-A2– recipient mice were then transplanted with mismatched allogeneic PB huCD34+cells (HLA-A3− HLA-A2+) at day 7 post-CD117×CD3 BTCE treatment (figure 5A). Mice were analyzed for huCD45+donor cell chimerism by STR analysis, the gold standard for chimerism quantification, 6 weeks post-transplantation. Based on 14 differential loci, we found 23%–89% (x±SEM: 65±15%) of CD45+HLA-A3−HLA-A2+ donor cells in the BM of transplanted CD117×CD3 BTCE treated mice (figure 5B, online supplemental table 1). Treatment with control HEL×CD3 BTCE resulted in no donor cell engraftment (data not shown). To gain more insights into the chimerism on the cellular level, we performed flow cytometric analyses. Confirming the STR analysis, we found 19%–89% (x±SEM: 63±16%) of huCD45+HLA-A3− HLA-A2+donor cells in the BM and 9%–57% (x±SEM: 40±14%) in PB, when animals were treated with CD117×CD3 BTCE but no chimerism with control BTCE (figure 5C–E, online supplemental figure 5A). Importantly, in CD117×CD3 BTCE-treated mice, donor HSC were functional and able to produce the expected hematopoietic cell types in the BM (figure 5F). These data show that the CD117×CD3 BTCE-mediated BM-conditioning protocol allows highly efficient allogenic HSCT without the need for chemo-mediated or irradiation-mediated ablation to create space within the BM niche.

### CD117×CD3 BTCE depletes AML and leukemia-initiating cells

**Figure 6:**
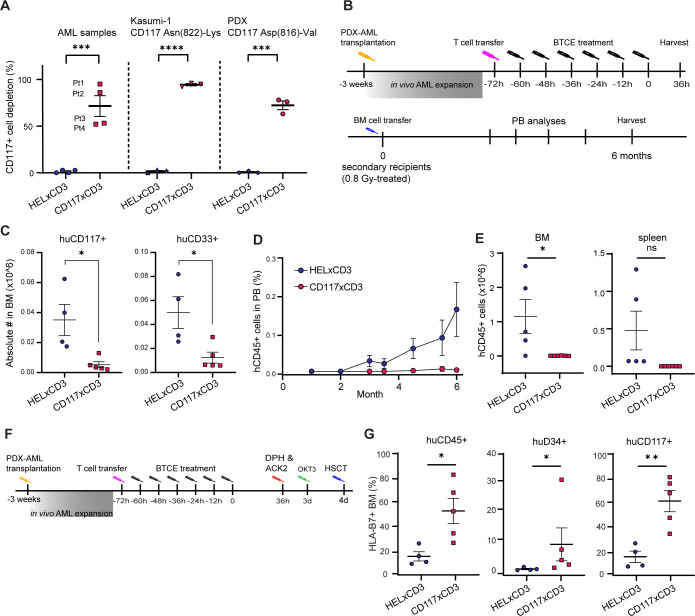
CD117×CD3 BTCE efficiently depletes AML and LICs. (**A**) Percentage of T cell-mediated depletion of indicated CD117+cells, when treated with 1.1 nM of indicated BTCE and co-cultured with human T cells (effector to target cell ratio of 4:1) for 24 hours (Kasumi-1) or 48 hours (primary AML samples and AML-PDX-CD117^ASP(816)-VAL^ in vitro) are shown. AML1 expresses the highest level of CD117, followed by AML2. AML3 and AML 4 express low CD117 levels. Data are representative of three experiments. (**B**) Schematic overview of the CD117×CD3 BTCE-mediated AML depletion and secondary recipient transplantation protocol of AML-PDX transplanted mice. (**C**) Absolute numbers of huCD117+AML PDX cells (left) and huCD33+AML PDX cells (right) in the BM of AML-PDX-transplanted mice (n=4 or 5 animals per group) at 36 hours post indicated BTCE treatments are shown. (**D**) NSG mice receiving BM from BTCE-treated AML-PDX animals. Percentages of huCD45+AML PDX cells in the PB of secondary transplanted NSG mice (n=6 animals per group) after indicated treatments and at specified time points are shown. (**E**) Absolute numbers of human CD45+cells in the BM (left) and spleen (right) of NSG mice (n=6 animals per group) at 6 months post-secondary BM transplantation depicted. (**F**) Schematic overview of AML-PDX depletion and allogenic HSCT protocol of AML-PDX mice. (**G**) Percentage of donor HLA-A3- HLA-B7+cells within the BM (left), huCD34+ (central) and huCD117+ (right) in the BM of AML-PDX mice (n=4 or 5 animals per group) after indicated treatments (**F**) and at 6 weeks post-HSCT. For figures A, C, E and G, the two-tailed Student’s t-test was used for statistical analysis. Lines (**A, C, E, G**) represent average, error bars in A, C, D, E, and G represent SEM. *p<0.05, **p<0.01, ***p<0.001, ****p<0.0001. AML, acute myeloid leukemia; BM, bone marrow; BTCE, bi-specific T-cell engagers; DPH, diphenhydramine hydrochloride; HSCT, hematopoietic stem cell transplantation; PB, peripheral blood; PDX, patient-derived xenograft.

Since 85% of patients with AML express CD117 on quiescent stem-like cells and proliferating stem/progenitor-like cells, cell-deleting immunotherapies that target CD117 may be a solution to eliminate AML with limited toxicity.^32 33^ First, we asked whether the level of CD117 on AML affects the CD117×CD3 BTCE-mediated killing of AML and selected four primary AML samples with various expression levels of CD117 (online supplemental figure 6A). Next, we performed in vitro killing assays and found that CD117×CD3 BTCE, but not HEL×CD3 efficiently killed CD117+AML cells in the presence of human HLA mismatched T cells. The CD117×CD3 BTCE-mediated killing was the most efficient with the highest expression of CD117 (AML1 and AML2), while control HEL×CD3 exhibited no AML killing (figure 6A).

To exclude that blocking of CD117 signaling and survival factor withdrawal, as observed with AMG191, is responsible for CD117×CD3 BTCE-mediated cytotoxicity, we performed in vitro killing assays with human Kasumi-1 cell line (CD117^Asn(822)-Lys^) and AML-patient-derived xenograft (PDX)-CD117^ASP(816)-VAL^ cells, which both carry an activating CD117 mutation. We observed high cytotoxicity with Kasumi-1 (95%), and AML-PDX-CD117^ASP(816)-VAL^ (72%) in vitro in the presence of human HLA mismatched T cells (figure 6A, online supplemental figure 6B,C). As expected, control CD117-U937 cells were not affected by CD117×CD3 BTCE (online supplemental figure 6D) and control HEL×CD3 BTCE exhibited no killing against Kasumi-1 and AML-PDX-CD117^ASP(816)-VAL^ cells (figure 6A). Together, our data indicate that CD117×CD3 BTCE efficiently depletes primary AML independent of CD117 signaling, as killing is not affected by constitutively active CD117 signaling.

LICs comprise a small fraction of the total leukemia cell population, are typically resistant to standard treatments and contribute to disease relapse.^34^ We therefore investigated whether CD117×CD3 BTCE can eradicate AML LICs in vivo. We generated AML-PDX-NSG mice using an AML-PDX line with normal karyotype and *FLT3-ITD*, a mutation that occurs in approximately 25% of AML cases. NSG mice were transplanted with AML-PDX cells of which 88% of the cells expressed CD117 (online supplemental figure 6E). As these mice have no human T cells, 3 weeks post AML-PDX transplantation, pre-activated human HLA mismatched T cells were first injected IP, and this was followed by six IP injections of CD117×CD3 BTCE (figure 6B). 36 hours post BTCE treatment, we observed a near complete depletion of human CD117+AML PDX cells in the BM of CD117×CD3 BTCE-treated mice compared with control BTCE-treated mice (figure 6C).

We then asked whether AML LICs were fully depleted in the CD117×CD3 BTCE-treated mice. Since the transfer of even a single LIC can generate AML in PDX mice,^35 36^ we transplanted BM cells of BTCE-treated AML-PDX mice into secondary NSG recipients and monitored for AML development for 6 months. We detected an increasing percentage of human AML-PDX cells in the PB of the mice transplanted with BM cells from HEL×CD3 BTCE-treated AML-PDX mice, and AML-PDX cells were also found in both BM and spleens at 6 months (figure 6D,E). In contrast, mice receiving BM from CD117×CD3 BTCE-treated AML-PDX mice exhibited undetectable AML-PDX cells in PB, BM and spleen up to 6 months post transplantation (figure 6D,E). The above shows CD117×CD3 BTCE efficiently depletes LICs below the level of leukemia initiation in mice secondarily transplanted with BM of treated mice. Based on the transfer of 2×10^5^ cells, this means that less than 1 leukemia-initiating unit per 200,000 BM cells remains after CD117×CD3 BTCE treatment, which prevents relapse in the long term.

### BM-conditioning of AML-PDX mice with CD117×CD3 BTCE allows efficient allogeneic HSCT

We next investigated whether CD117×CD3 BTCE treatment of AML-PDX mice allows efficient allogeneic HSCT. Therefore, AML-PDX (HLA-A3+HLA-B7−) mice were transplanted with mismatched human PB CD34+cells (HLA-A3− HLA-B7+) 4 days post-CD117×CD3 BTCE treatment. Mice also received pre-activated human T cells, ACK2 and OKT3 antibodies (figure 6F). 6 weeks post-transplantation, CD117×CD3 BTCE-treated mice showed up to 80% HSPC chimerism (27%–83, x±SEM: 53±12%) of human donor cells (CD117+HLA-A3− HLA-B7+), whereas the donor cell chimerism in HEL×CD3 BTCE-treated mice was significantly lower (11%–26%, x±SEM: 15±4) (figure 6G). Altogether, these data indicate that CD117×CD3 BTCE potently depletes LICs and allows efficient allogenic HSCT in leukemia-bearing mice.

## Discussion

In this study, we have generated and tested a CD117×CD3 BTCE for rapid and effective BM conditioning and depletion of bulk leukemic cells and LICs prior to HSCT. We showed that CD117×CD3 BTCE binds specifically to human CD117+ and CD3+ cells. We demonstrated that the binding of CD117×CD3 BTCE to CD3 in the absence of targets is not sufficient to trigger the activation of T cells and the secretion of cytokines. Vice versa, human CD117+target cells were not eliminated by CD117×CD3 BTCE in the absence of T cells, indicating that depletion by CD117×CD3 BTCE is not due to blocking of SCF-mediated CD117 survival signaling. This was further confirmed by demonstrating CD117×CD3 BTCE-mediated killing of AML cells with constitutive active CD117 signaling. We confirmed a highly efficient CD117×CD3 BTCE-mediated depletion of HSPCs and LICs in preclinical mouse models, while CD117×CD3 BTCE induced only a transient low level of inflammatory cytokines in humanized NSG mice. The highly efficient target cell-depleting activity and the absence of detrimental consequences of the CD117×CD3 BTCE treatment in mice make this reagent promising for further clinical development.

A different anti-CD117 reagent, briquilimab (JSP191), has already been tested as a single reagent or in combination with low-dose irradiation and fludarabine conditioning in a clinical trial for the treatment of patients with minimal residual disease-positive MDS/AML undergoing allogeneic HSCT (NCT04429191), sickle cell disease (NCT05357482), Fanconi anemia (NCT04784052) and SCID patients (NCT02963064). Although the results of these studies suggested the safety and feasibility of the treatment, improvements in donor cell chimerism after HSCT and patient outcomes are still needed. For instance, there is a high risk of disease relapse and progression in both AML (3/13) and MDS (5/16) patients in 1 year, and 18 out of 32 patients developed graft versus host disease.^37^ In addition, in SCID patients the donor cell chimerism is poor, most likely due to prolonged activity of the anti-CD117 antibodies of up to 3 weeks, which allows recovery of the diseased BM CD34+cell population before HSCT (NCT02963064). The CD117×CD3 BTCE-mediated conditioning may improve BM-conditioning and HSCT due to its highly efficient HSC-depleting activity and very short serum half-life of~2 hours. This half-life is significantly longer compared with the CD117×CD3 BTCE reagent recently published by Volta *et al*, which has a half-life of approximately 30 min,^22^ something that may be important for clinical treatment protocols. Despite the short serum half-life, our data showed that CD117×CD3 BTCE can be detected on T cells up to 7 days due to the high-affinity anti-CD3 arm, a potential hurdle for some clinical applications, that is not investigated by Volta *et al*.^22^ This may hinder HTSC but can be circumvented with conditioning therapies that eradicate remaining CD117×CD3 BTCE-coated T cells. Such therapies are already used prior to allogeneic transplantation and use cyclophosphamide or T-cell-depleting antibodies. Other solutions could be a BTCE with a lower affinity anti-CD3 arm or a BTCE purging strategy. Whether this affects the efficiency of CD117+HSPC and LIC depletion remains to be determined.

Whether CD117+cell depletion with the CD117×CD3 BTCE reagent of Volta *et al* allows donor HSC engraftment in the BM after HSCT remains to be investigated. We addressed this question and found that the highly efficient depletion of hu-CD34 (>99%) allows for donor HuCD34 cell engraftment in humanized mice. However, chimerism achieved was lower than expected from such deep depletion of recipient hu-CD34 cells. This is probably due to donor cells in our system being challenged by suboptimal homing to the mouse BM niche, a microenvironment that diverges functionally from human BM. Additionally, the residual Hu-CD34+cells are pre-adapted to the murine BM niche, conferring a competitive growth advantage over newly infused donor cells. These limitations in humanized mice suggest that in the human setting an even higher level of donor cell chimerism may be expected with our CD117×CD3 BTCE. Together, our results suggest that patients may receive allogeneic donor cells rapidly after CD117×CD3 BTCE treatment and T-cell depletion, which will presumably improve the HSCT efficiency and decrease the risk of relapse compared with antibody-based conditioning protocols.

Despite the absence of clear CD117×CD3 BTCE-induced toxicity in NSG mice, the obtained results are not fully conclusive due to lack of cross-reactivity with mouse CD117-expressing tissues. CD117 is expressed on many different cell types in humans. For instance, CD117 is expressed on limbal melanocytes and sweat glands in the skin, tubular epithelial cells in the kidney, melanocytes and retinal glia cells in the eye.^38^,^41^ Hence, the risk for CD117×CD3 BTCE-induced tissue damage remains to be investigated in clinically relevant models such as non-human primates. Although the potential risk of BTCE treatments for patients needs to be further evaluated, there are several indications from recent clinical investigations showing that CD117 targeting is safe in humans. First, briquilimab (JSP191, AMG191) anti-CD117 antibodies did not cause obvious toxicities in non-human primates^24^ and more than 110 healthy volunteers and patients in clinical trials (NCT04429191, NCT02963064, NCT04784052, NCT05357482) undergoing allogeneic HSCT. Only in a clinical trial in patients with AML and MDS targeting CD117 with MGTA-117 a novel antibody drug conjugate with amanitin, an RNA polymerase II inhibitor, unacceptable toxicity was observed and this was most likely due to the amanitin moiety (NCT05223699). Together, these data indicate that the transient depletion of the various CD117+cell populations is not detrimental for the patients.

Clinical investigation of BTCE treatments in various types of hematopoietic malignancies, including B-cell acute lymphoblastic leukemia, multiple myeloma and AML, has demonstrated compelling results. Increasing numbers of US Food and Drug Administration-approved T-cell engagers for targeting, for example, CD19 (blinatumomab, Blincyto),^42^ BCMA (teclistamab and elranatamab),^43 44^ CD20 (mosunetuzumab, epcoritamab and glofitamab)^45^,^47^ and GPRC5D (talquetamab)^48^ are now available. However, cytokine release syndrome (CRS), characterized by fever and constitutional symptoms (grade 1) to life-threatening hypoxia and hypotension (grade 4), is a common BTCE-related toxicity and needs monitoring during treatments.^49^ CRS usually occurs in the first cycle of the treatment, and is mostly mild (grade 1–2), well-treatable with corticosteroids or drugs that block cytokines or by decreasing BTCE doses, and therefore manageable in most cases. Although a high affinity of the CD3 part of BTCE is important for proper T-cell activation and cytotoxicity, a weaker CD3 arm may reduce the risk for CRS.^50^ Despite the lack of CD117×CD3 BTCE-induced CRS in huCD34-NSG mice, the obtained results are not conclusive due to lack of cross-reactivity with mouse CD117 and reduced CRS-inducing innate immune cell types, including macrophages and monocytes, in these mice and this needs further investigation in non-human primates and clinical safety studies. The reagent described by Volta *et al*, used the CD117 binding sequence of the 79D antibody and the anti-CD3 sequence of OKT3, which is restricted to human T-cell recognition and precludes toxicology studies in non-human primates. An advantage of our combination of antibody sequences used for the CD117×CD3 BTCE (hSR1 for targeting CD117 and SP34-2 for targeting CD3) is that our reagent cross-reacts with CD117 and CD3 molecules of non-human primates such as cynomolgus monkeys. This allows further preclinical testing of our CD117×CD3 BTCE to test its efficiency and safety. The latter is critical, because despite our reagent being safe in humanized mice, toxicity can only be fully evaluated in non-human primates. This is due to our reagent not binding mouse CD117 and thus we cannot fully assess on-target toxicities in mice.

In our experimental set-up we show that with our protocol, CD117×CD3 BTCE sufficiently eradicates LICs below the level of leukemia initiation, that is, less than 1 leukemia-initiating unit in 200,000 BM cells in an immune-compromised mouse within 6 months. Although our data present strong evidence for deep CD117×CD3 BTCE-mediated LIC depletion, whether CD117×CD3 BTCE prevents AML minimal residual disease and relapse in the clinical setting remains to be determined.

Taken together, we describe a CD117×CD3 BTCE that effectively depletes human CD117+HSPCs and LICs and allows robust human donor HSC engraftment in humanized mouse models. Our data provide support for further development of CD117×CD3 BTCE-mediated conditioning and testing of CD117×CD3 BTCE in non-human primates with the desired goal to improve the safety of LIC depletion and BM-conditioning, enhance the efficiency of donor cell engraftment and simultaneously eliminate the risk for toxicities in the short- and long-term. The expectation of rapid development in the near future of safe gene transfer or editing strategies for monogenic diseases that affect hematopoietic cells makes the need to develop safe and efficient HSCT modalities more urgent. The BTCE approach we describe holds such promise of safe and efficient BM conditioning.

## Supplementary material

10.1136/jitc-2025-011888 online supplemental file 1

10.1136/jitc-2025-011888 online supplemental file 2

## Data Availability

Data are available upon reasonable request.
