# Supplementary material for: Immunotherapy for rapid bone marrow conditioning and leukemia depletion that allows efficient hematopoietic stem cell transplantation
Source: J Immunother Cancer. 2025 Jun 27;13(6):e011888. doi: 10.1136/jitc-2025-011888 (PMC12207149; doi:10.1136/jitc-2025-011888)
Supplement: online supplemental file 1 [file jitc-13-6-s001.pdf]

Highly efficient and safe hematopoietic stem cell transplantation for benign and malignant diseases

Giada Dal Collo, et al.

Supportive data

Supplementary Figure 1

Sequence of CD117xCD3 BTCE

Chain_1
DIVMTQSPDSLAVSLGERATINCRASESVDIYGNSFMHWYQQKPGQPPKLLIYLASNLES
GVPDRFSGSGSGTDFTLTISSLQAEDVAVYYCQQNNEDPYTFGGGTKVEIKRTVAAPSVF
IFPPSDEQLKSGTASVVCLLNNFYPREAKVQWKVDNALQSGNSQESVTEQDSKDSTYSLS
STLTLSKADYEKHKVYACEVTHQGLSSPVTKSFNRGECEVQLVESGGGLVQPGGSLKLSC
AASGFTFNTYAMNWVRQASGKGLEWVGRIRSKYNNYATYYADSVKDRFTISRDDSKSTLY
LQMNSLKTEDTAVYYCVRHGNFGNSYVSWFAYWGQGTLVTVSSASTKGPSVFPLAPSSKS
TSGGTAALGCLVKDYFPEPVTVSWNSGALTSGVHTFPAVLQSSGLYSLSSVVTVPSSSLG
TQTYICNVNHKPSNTKVDKKVEPKSC

Chain_2
QVQLVQSGAEVKKPGASVKVSCKASGYTFTSYNMHWVRQAPGQGLEWMGVIYSGNGDTSY
NQKFKGRVTITADKSTSTAYMELSSLRSEDTAVYYCARERDTRFGNWGQGTLVTVSSAST
KGPSVFPLAPSSKSTSGGTAALGCLVKDYFPEPVTVSWNSGALTSGVHTFPAVLQSSGLY
SLSSVVTVPSSSLGTQTYICNVNHKPSNTKVDKKVEPKSC

Chain_3
QAVVTQEPSLTVSPGGTVTLTCRSSTGAVTTSNYANWVQQKPGQAPRGLIGGTNKRAPWT
PARFSGSLLGDKAALTLLGAQPEDEAEYFCALWYSNLWVFGGGTKLTVLGQPKAAPSVTL
FPPSSEELQANKATLVCLISDFYPGAVTVAWKADSSPVKAGVETTTPSKQSNNKYAASSY
LSLTPEQWKSHRSYSCQVTHEGSTVEKTVAPTECS

Sequence of HELxCD3 BTCE

Chain_1
DIVLTQSPAIMSASPGEKVTMTCSASSSVNYMYWYQQKSGTSPKRWIYDTSKLASGVPVR
FSGSGSGTSYSLTISSMETEDAAEYYCQQWGRNPTFGGGTKLEIKRTVAAPSVFIFPPSD
EQLKSGTASVVCLLNNFYPREAKVQWKVDNALQSGNSQESVTEQDSKDSTYSLSSTLTLS
KADYEKHKVYACEVTHQGLSSPVTKSFNRGECEVQLVESGGGLVQPGGSLKLSCAASGFT
FNTYAMNWVRQASGKGLEWVGRIRSKYNNYATYYADSVKDRFTISRDDSKSTLYLQMNSL
KTEDTAVYYCVRHGNFGNSYVSWFAYWGQGTLVTVSSASTKGPSVFPLAPSSKSTSGGTA
ALGCLVKDYFPEPVTVSWNSGALTSGVHTFPAVLQSSGLYSLSSVVTVPSSSLGTQTYIC
NVNHKPSNTKVDKKVEPKSC

Chain_2
EVQLQQSGAELMKPGASVKISCKASGYTFSDYWIEWVKQRPGHGLEWIGEILPGSGSTNY
HERFKGKATFTADTSSSTAYMQLNSLTSEDSGVYYCLHGNYDFDGWGQGTTLTVSSASTK
GPSVFPLAPSSKSTSGGTAALGCLVKDYFPEPVTVSWNSGALTSGVHTFPAVLQSSGLYS
LSSVVTVPSSSLGTQTYICNVNHKPSNTKVDKKVEPKSC

Chain_3
QAVVTQEPSLTVSPGGTVTLTCRSSTGAVTTSNYANWVQQKPGQAPRGLIGGTNKRAPWT
PARFSGSLLGDKAALTLLGAQPEDEAEYFCALWYSNLWVFGGGTKLTVLGQPKAAPSVTL
FPPSSEELQANKATLVCLISDFYPGAVTVAWKADSSPVKAGVETTTPSKQSNNKYAASSY
LSLTPEQWKSHRSYSCQVTHEGSTVEKTVAPTECS

4

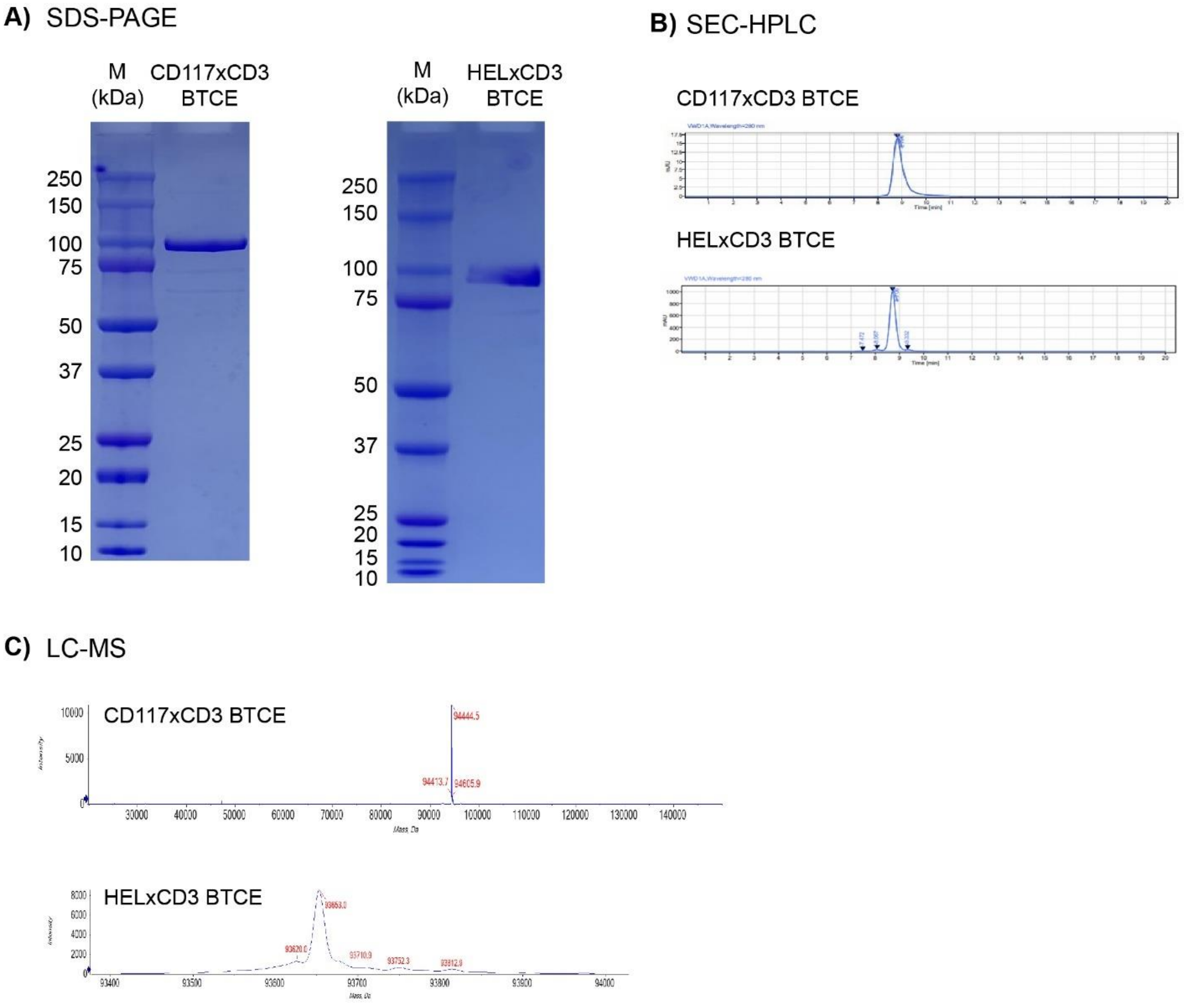

Supplementary Figure 2. Expression and characterization of CD117xCD3 BTCE. A) CD117xCD3 BTCE and HELxCD3 BTCE exhibited a migration at the expected size of ~95 kDa in SDS-PAGE gel under non-reducing conditions. **B)** SEC-HPLC analyses showed the purity of the reagent. **C)** LC-MS analyses of the purified CD117xCD3 BTCE and HELxCD3 BTCE showed a mass of 94,444.5 Da. and 93653.0 Da respectively. M: Marker, kDa: kilo Dalton , SDS-PAGE: sodium dodecyl sulfate polyacrylamide gel electrophoresis, SEC-HPLC: Size Exclusion Chromatography-High-performance liquid chromatography. LC-MS: Liquid chromatography-Mass Spectrometry.

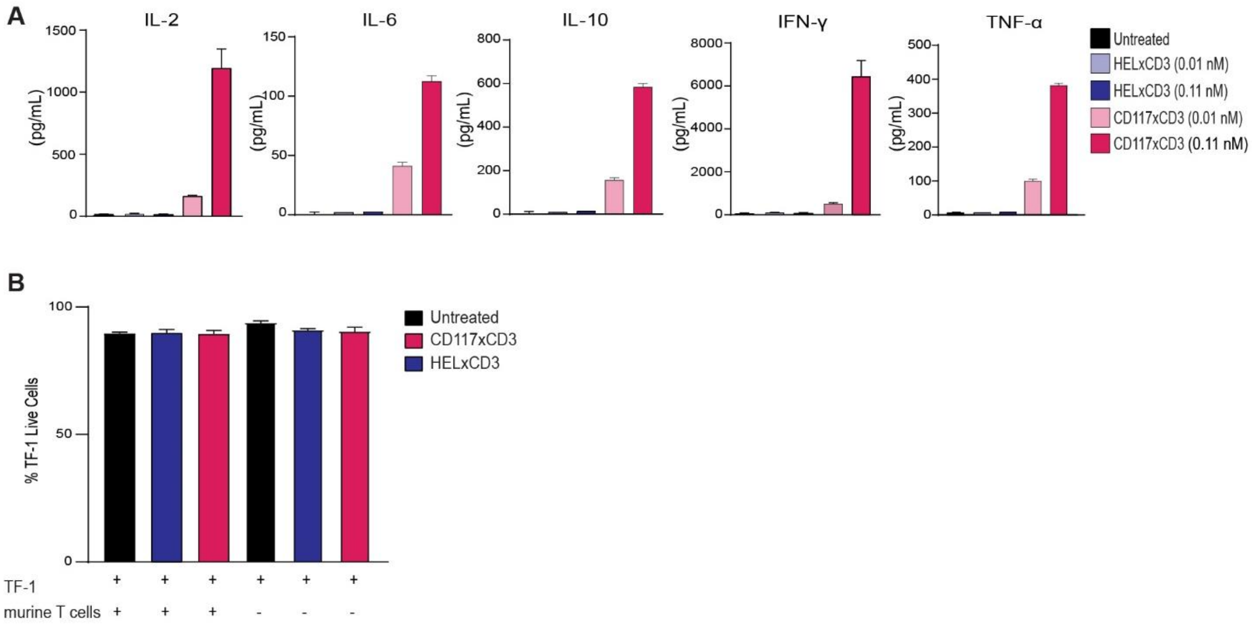

Supplementary Figure 3. ***In vitro*** **CD117xCD3 BTCE-mediated T cell activation. (A)** Concentrations of inflammatory cytokines determined in the supernatant of TDCC assays with indicated BTCE and TF-1 target cells are shown. Results are representative of 3 experiments. **(B)** Percentage of live TF-1 target cells when treated with BTCEs (11.1 nM) and co-cultured with murine T cells (E:T ratio of 4:1) for 24 hours *in vitro* are shown. Results are representative of 3 experiments. Error bars in A and B indicate SEM of technical triplicates.

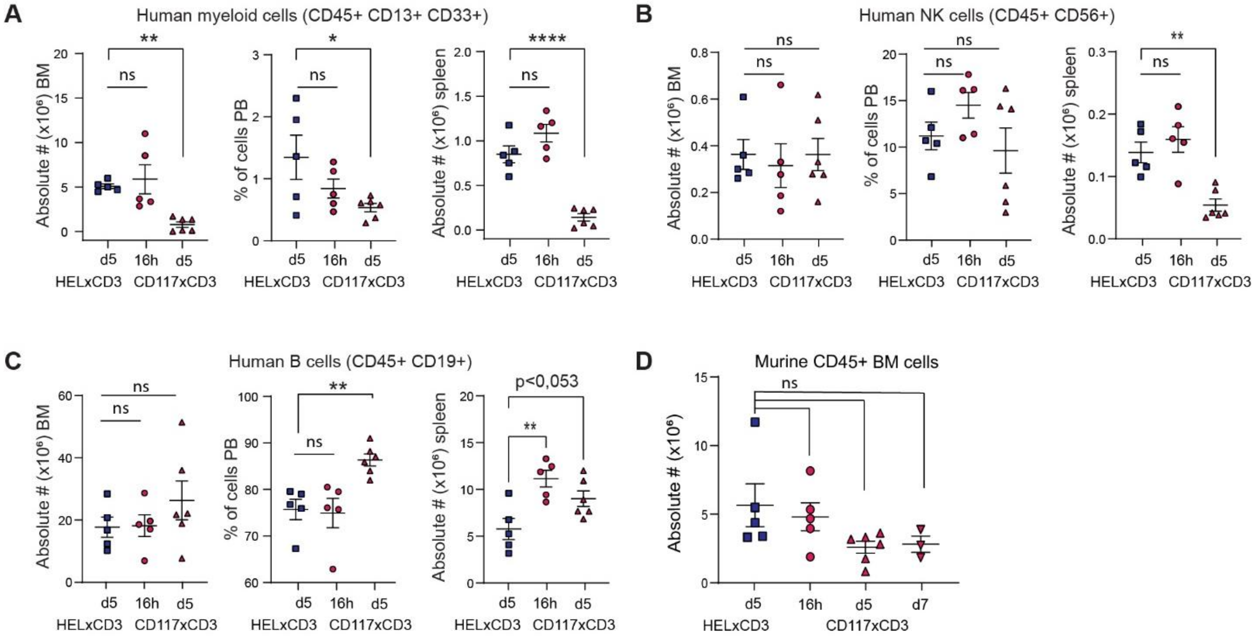

Supplementary Figure 4. BTCE treatment affects cytokine levels and various cell populations in the BM of huCD34-NSG mice. (A) Absolute numbers of human myeloid cells (CD45+ CD13+ CD33+) in the BM (left), spleen (right) or percentage of myeloid cells in the PB (middle) of humanized mice (n=5 or 6 animals per group) after indicated treatment and at specified time points are shown. **(B)** Absolute numbers or percentage of NK cells (CD45+ CD56+) in the BM (left), PB (middle) and spleen (right) of humanized mice (n=5 or 6 animals per group) after indicated treatment and at specified time points are presented. **(C)** Absolute numbers of B cells (CD45+ CD19+) in BM (left), spleen (right) and percentage of B-cells in the PB (middle) of humanized mice (n=5 or 6 animals per group) after indicated treatment and at specified time points are shown. **(D)** Absolute numbers of mouse CD45+ cells in the BM of humanized mice (n= 5 or 6 animals per group) after indicated treatment and at specified time points are shown. For figures A, B, C and D, a one-way ANOVA with Dunnett's multiple comparisons test was used for statistical analysis. Error bars represent SEM. $^*p < 0.05$, $^{**}p < 0.01$, $^{***}p < 0.001$. ns: not significant.

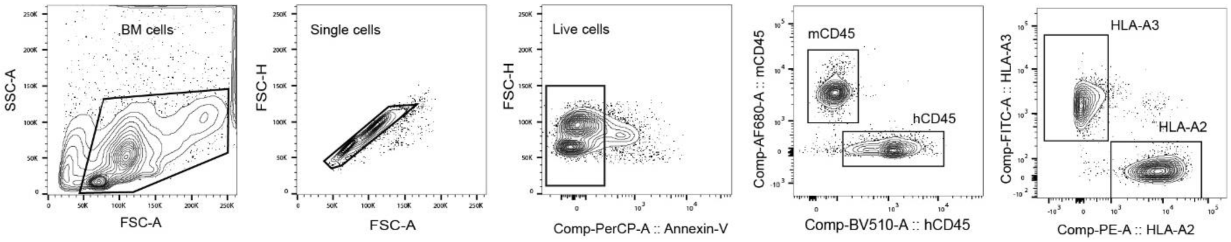

Supplementary Figure 5. Gating strategy. Representative FACS plots showing the gating strategy of human CD45+/HLA-A2+ donor and CD45+/HLA-A3+ recipient cells in the BM of BTCE-treated huCD34-NSG mice.

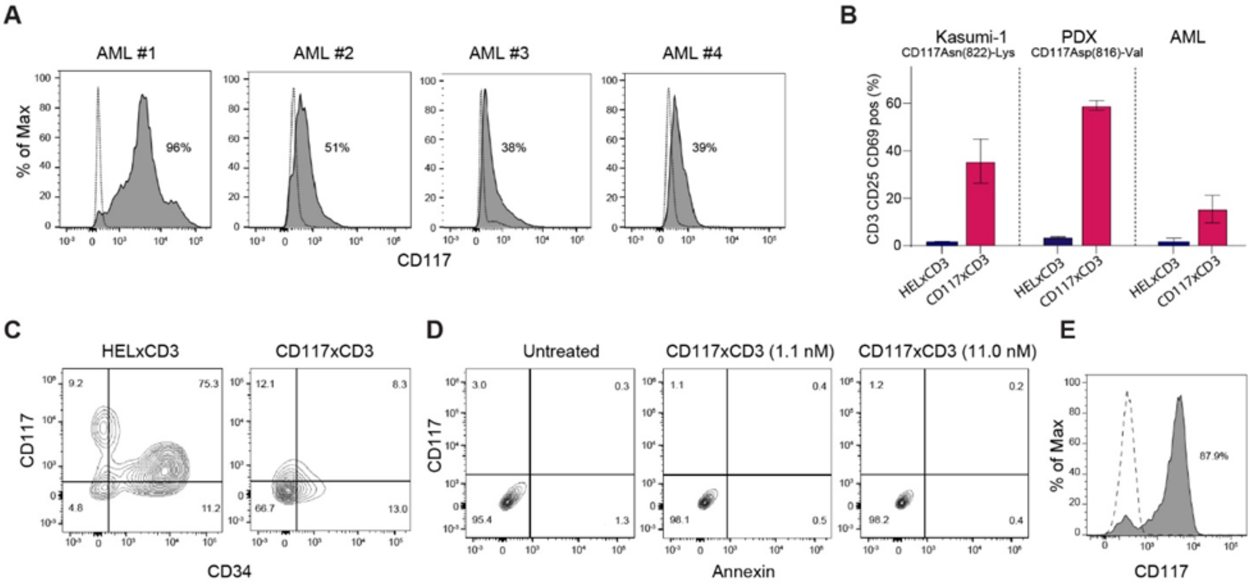

Supplementary Figure 6. CD117xCD3 BTCE-mediated T cell activation and AML depletion. (A) FACS plots showing CD117 expression on four selected AML samples. Samples tested are from adult AML patients at diagnosis expressing CD117. The molecular information of the AML patient cells are: Sample 1: NPM1 mutation and in-frame bZIP mutated CEBPA, Sample 2: NPM1 mutation, Sample 3: AML cells contain mutated form of NPM1, IDH1, PTPN11 and FLT3-TKD, Sample 4: AML cells contain mutations in ASXL1, IDH1, EZH2, and STAG2. **(B)** Percentage of CD25+ CD69+ T cells after co-culture with Kasumi-1 (CD117$^{Asn(822)-Lys}$), AML-PDX-CD117$^{Asp(816)-Val}$ and primary AML samples (n=4) in the presence of indicated BTCE are shown. Experiments were performed in triplicate and presented data are representative for at least 3 experiments. Error bars represent SEM. **(C)** Representative FACS plots showing CD117+ CD34+ AML cells after treatment with indicated BTCE. **(D)** Representative FACS plots showing the absence of Annexin V+ signals on CD117-negative U937 AML cells after indicated BTCE treatment. **(E)** Representative FACS plots showing CD117 expression on AML-PDX cells.

Supplementary Table 1: Donor cell chimerism in the BM of transplanted BTCE-treated huCD34-NSG mice

	Mouse #1	**Mouse #2**	**Mouse #3**	**Mouse #4**
**STR Loci**	**%D CHM**			
D8S1179	82.9%	69.1%	20.8%	92.2%
D21S11	78.3%	62.4%	23.8%	81.3%
D7S820	82.2%	68.7%	24.2%	88.9%
CSF1PO	84.1%	67.7%	32.5%	98.7%
D3S1358	NI	NI	NI	NI
TH01	80.0%	69.3%	26.5%	85.1%
D13S317	93.0%	82.1%	25.1%	NI
D16S539	83.9%	61.7%	22.3%	87.0%
D2S1338	73.6%	62.1%	18.1%	88.7%
D19S433	NI	NI	NI	NI
vWA	77.7%	63.7%	19.5%	83.5%
TPOX	78.6%	61.3%	18.4%	91.5%
D18S51	78.0%	68.9%	22.8%	88.4%
AMEL	77.0%	67.7%	22.9%	91.8%
D5S818	75.7%	62.5%	22.2%	85.5%
FGA	77.9%	64.9%	20.0%	89.1%
Average %Donor CHM	**80.2%**	**66.6%**	**22.8%**	**88.6%**
Standard Deviation	4.8	5.4	3.7	4.5
Coefficient of Variation	6.0%	8.2%	16.3%	503.0%
Margin of Error	2.8% (95%)	3.1% (95%)	2.2% (95%)	2.7% (95%)
Number of Informative Loci	14	14	14	13

The percentage of donor chimerism of each STR locus identified in the BM cells of CD117xCD3 BTCE-treated HuCD34-NSG mice (n=4) at 6 weeks post-HSCT are shown. The average percentage of donor cell chimerism of 14 (13 for mouse 4) out of 16 different loci analyzed are indicated in red. NI = not identified, %D CHM = percentage of donor chimerism.

Supplementary Table 2: Overview of antibodies used for flow cytometry

Antigen/marker	*Label*	*Species*	*Clone #*	*Supplier*	*Catalogue #*	*Dilution*
Annexin V	APC	-	-	BD	550475	1:20
Annexin V	BV421	-	-	BD	563973	1:40
Annexin V	PerCP-Cy5.5	-	-	BD	561431	1:80
CD3	BV421	human	UCHT1	BD	562426	1:40
CD3	FITC	human	UCHT1	BD	561806	1:20
CD3	PerCP-Cy5.5	human	UCHT1	BD	560835	1:40
CD3	PE-CF594	human	UCHT1	BD	562280	1:160
CD4	PE-CF594	mouse	RM4-5	BD	562285	1:400
CD4	BV650	human	SK3	BD	563875	1:40
CD8a	BV711	mouse	53-6.7	BD	563046	1:40
CD8	BV786	human	RPA-T8	BD	563823	1:80
CD13	PE	human	WM15	BioLegend	301703	1:160
CD14	PE-Cy7	human	61D3	eBioscience	E10278-1637	1:160
CD16	BV510	human	3G8	BD	563830	1:20
CD19	PerCP-Cy5.5	human	HIB19	eBioscience	45-0199-42	1:20
CD25	SB600	human	BC96	Invitrogen	63-0259-42	1:20
CD33	PE	human	WM53	BioLegend	303403	1:160
CD34	APC	human	561	BioLegend	343607	1:40
CD34	PE-Cy7	human	561	BioLegend	343615	1:80
CD38	PE-Cy7	human	HIT2	BioLegend	303515	1:10
CD45	AF700	mouse	30-F11	BioLegend	103128	1:20
CD45	APC-Cy7	mouse	30-F11	BioLegend	103115	1:40
CD45.1	FITC	mouse	A20	BioLegend	110706	1:20
CD45	APC-Cy7	human	HI30	BioLegend	304014	1:50
CD45	BV510	human	HI30	BioLegend	304036	1:20
CD45	FITC	human	HI30	BioLegend	304006	1:20
CD45RA	APC-H7	human	HI100	BD	560674	1:40
CD56	BV785	human	5.1H11	BioLegend	362550	1:40
CD56	PE-CF594	human	B159	BD	562289	1:20
CD69	BV711	human	FN50	BD	563836	1:20
CD90	PE	human	5 E10	BioLegend	328109	1:10
CD117	BV421	human	104D2	BioLegend	313216	1:20
Lineage	BV510	human	OKT3	BioLegend	348807	1:10
HLA-A2	PE	human	BB7.2	BD	558570	1:20
HLA-A3	FITC	human	GAP.A3	Invitrogen	11-5754-42	1:20
HLA-B7	APC	human	BB7.1	Invitrogen	372406	1:20
Hoechst 33342	DAPI	-	-	Invitrogen	R37165	-

Supplementary Table 3: Antibodies used for high-dimensional flow cytometry

Antigen/Marker	Label	Species	Clone #	Supplier	Catalogue #	µl/ stain
CD45RA	BUV395	human	5H9	BD	740315	1.2
Annexin V	AF350	-	-	ThermoFisher	A23202	1
CD16	BUV496	human	3G8	BD	612944	0.6
CD195 CCR5	BUV563	human	2D7/CCR5	BD	741401	2.5
CD314	BUV615	human	1D11	BD	751232	5
CD39	BUV661	human	TU66	BD	749967	2.5
CD56	BUV737	human	NCAM16.2	BD	564447	1.2
CD8	BUV805	human	SK1	BD	612889	1.2
CD197 CCR7	BV421	human	G043H7	BioLegend	353208	5
CD123	SB436	human	6H6	ThermoFisher	62-1239-42	2.5
CD11c	eF450	human	3.9	ThermoFisher	48-0116-42	2.5
IgD	BV480	human	IA6-2	BD	566138	0.6
CD3	BV510	human	SK7	BioLegend	344828	2.5
CD20	PacOrange	human	HI47	ThermoFisher	MHCD2030	4.0
IgM	BV570	human	MHM-88	BioLegend	314517	2.5
IgG	BV605	human	G18-145	BD	563246	7.0
CD28	BV650	human	CD28.2	BioLegend	302946	2.5
CD196 CCR6	BV711	human	G034E3	BioLegend	353436	1.2
CD185 CXCR5	BV750	human	RF8B2	BD	747111	1.2
CD279 PD-1	BV785	human	EH12.2H7	BioLegend	329930	5
CD141	BB515	human	1A4	BD	565084	2.5
CD57	FITC	human	HNK-1	BioLegend	359604	0.5
CD14	SparkBlue550	human	63D3	BioLegend	367148	1.25
CD45	PerCP	human	HI30	ThermoFisher	MHCD4531	1.2
CD2	PerCP-Cy5.5	human	TS1/8	BioLegend	309226	5
γδTCR	PerCPeF710	human	B1.1	ThermoFisher	46-9959-42	2.5
NKG2c CD159c	PE	human	REA205	Miltenyi	130-119-776	2
CD4	cFluorYGF584	human	SK3	Cytek	R7-20041	1.2
CD337	PE-Dazzle594	human	P30-15	BioLegend	325232	5
CD24	PE-AF610	human	SN3	ThermoFisher	MHCD2422	2.5
CD95	PE-Cy5	human	DX2	ThermoFisher	15-0959-42	0.6
CD25	PE-AF700	human	CD25-3G10	ThermoFisher	MHCD2524	2.5
CD183 CXCR3	PE-Cy7	human	G025H7	ThermoFisher	25-1839-42	2.5
HLA-DR	PE-Fire810	human	L243	BioLegend	307683	0.6
CD45	AF700	mouse	30-F11	BioLegend	103128	1
CD1c	AF647	human	L161	BioLegend	331510	5
CD19	SparkNIR685	human	HIB19	BioLegend	302270	1.2
CD127	APC-R700	human	HIL-7R-M21	BD	565185	4
CD159a	APC	human	REA110	Miltenyi	130-113-563	2
CD27	APC-H7	human	M-T271	BD	560222	2.5
CD38	APC-Fire810	human	HIT2	BioLegend	303550	1
