# Supplementary material for: Immunotherapy for rapid bone marrow conditioning and leukemia depletion that allows efficient hematopoietic stem cell transplantation
Source: J Immunother Cancer. 2025 Jun 27;13(6):e011888. doi: 10.1136/jitc-2025-011888 (PMC12207149; doi:10.1136/jitc-2025-011888)
Supplement: online supplemental file 2 [file jitc-13-6-s002.pdf]

Supplementary Material & Methods

Generation of the CD117xCD3 BTCE engaging and activating antibody-construct

Both CD117xCD3 and HELxCD3 BTCEs were generated in the format of Fab-Fab stacking, which consists of three polypeptide chains: Chain 1: TAA_VK-CK-CD3_VH-CH1, Chain 2: TAA_VH-CH1 and Chain 3: CD3_VL-CL. CK is human kappa (κ) constant domain; CH1 is human IgG1 CH1 constant domain; CL is human lambda (λ) constant domain; TAA_VK is light chain variable domain (κ) of anti-TAA where TAA could be CD117 or HEL (hen egg lysozyme); TAA_VH is heavy chain variable domain of anti-TAA where TAA could be CD117 or HEL; CD3_VH is heavy chain variable domain of anti-CD3; CD3_VL is light chain variable domain (λ) of anti-CD3. The VH and VK sequences of anti-CD117 are derived from CD117 antibody AMG-191 (hSR1) (Ng G, Shen W, inventors; Amgen Inc, assignee. Humanized c-Kit antibody. US patent 7,915,391 B2. 29 March 2011). The VH and VL sequences of anti-CD3 are humanized variants derived from anti-CD3e clone SP34-2 (1). The VH and VK sequences of anti-HEL are derived from clone HYHEL5 published in Abysis database (http://www.abysis.org/) with accession numbers 001784 and 006278, respectively. The transient production for CD117xCD3 and HELxCD3 BTCE were following regular procedure, briefly, three plasmids encoding three polypeptide chains were co-transfected to mammalian expression host cells e.g. expi-CHO-s, then after culturing for 7-9 days, the supernatants were harvested and the antibodies in supernatants were purified by protein A affinity chromatography and followed by further polishing step with Mono-S cation exchange column (Cytiva). Antibody reagents were validated with PAGE, SEC-HPLC and LC-MS according to standard protocols.

Cell Culture

All human cell lines were grown in complete medium A (RPMI 1640 medium supplemented with fetal bovine serum [FBS, 10%, Gibco] [Kasumi-1 cells 20% FBS], L-Glutamine [2 mM, Life Technologies], penicillin [100 U/mL, Gibco] and Streptomycin-sulfate [100 µg/mL, Gibco], Sodium Pyruvate [1 mM, Life Technologies]). For the expansion of TF-1 cells, human granulocyte-macrophage colony-stimulating factor (GM-CSF, 5 ng/mL, Peprotech) was added to the medium. In general, cells were maintained at a concentration of 1-2x10^5 cells/mL in a humidified incubator with CO_2 (5%) at 37°C. All cell lines were passaged less than 3 months after thawing.

Primary human CD34+ purified cells from bone marrow (BM CD34+ cells) and peripheral blood (PB CD34+ cells) were purchased from StemCell Technologies and obtained from our internal transplantation laboratory (Erasmus MC, the Netherlands), respectively. For *in vitro* HSC assay complete medium B (serum-free StemSpan SFEM II media, supplemented with stem cell factor [SCF] [100 ng/mL], thrombopoietin [TPO] [100 ng/mL], Flt3 ligand [100 ng/mL] [all from Peprotech, NJ, USA], UM729 [1 µM] [stem cell technologies] and SR-1 [0.5 µM] [stem cell technologies]) was used.

Human peripheral blood mononuclear cells (PBMCs) were isolated from healthy donors (Sanquin, Netherlands) by Ficoll density centrifugation (GE Healthcare, IL, USA). Human CD3+ T cells were purified from frozen human PBMCs using RosetteSep™ Human T Cell Enrichment Cocktail (StemCell Technologies), according to the manufacturer's instructions. Purified human T cells were plated in a 48-wells plate at a concentration of 1×10^6 cells/mL in complete medium C (RPMI 1640 medium supplemented with FBS [10%], L-Glutamine [2 mM], penicillin [100 U/mL],

Streptomycin-sulfate [100 µg/mL] and human Interleukin-2 [hIL-2, 20 UI/mL, Roche]) and incubated overnight for recovery.

The PDX cells harboring t(8;21) ([AML-PDX-CD117$^{Asp(816)-Val}$], kindly provided by prof. dr. Olaf Heidenreich, Princess Máxima Center for Pediatric Oncology) were cultured in serum free condition with SFEMII medium supplemented with penicillin/streptomycin (100 U/mL), IL-3 (10 ng/mL), FLT3 ligand (10 ng/mL), GM-CSF (10 ng/mL), SCF (150 ng/mL), TPO (100 ng/mL), SR1 (750 nM), and UM729 (1.35 µM). AML samples were maintained in culture for 3 days for recovery prior to initiating the experiment.

Flow Cytometry Staining

All the fluorochrome-conjugated antibodies listed in **Supplementary Table 2** were used to stain the *in vitro* or *ex vivo* harvested cells for flow cytometry. In case of whole blood samples (20-50 µL) from mice, red blood cells were first lysed in 2 mL of Ammonium Chloride Potasium (ACK) lysis buffer (NH4Cl [150 mM], KHCO3 [10 mM], EDTA [0.1 mM]) on ice for 2 minutes. For staining of surface antigens, cells were washed with FACS wash buffer (HBSS containing 3% FBS and 0.02% sodium azide) and incubated with Fc receptor blocking antibody (Human TruStain FcX™, Biolegend), followed by incubation of pre-determined optimal concentrations of fluorochrome-conjugated monoclonal antibodies at 4 °C in the dark for 20 minutes. To exclude apoptotic and dead cells, Annexin-V conjugated with either APC, BV421 or PerCP-Cy5.5 and calcium chloride (CaCl2, 2.5 mM) were added to the staining solutions. After staining, cells were washed with FACS wash buffer and fixed with paraformaldehyde (1% v/v) + CaCl2 (2.5 mM). Samples were acquired on a BD LSRII-Fortessa flow cytometer system (BD Biosciences), with FACS Diva software.

Alternatively, to exclude apoptotic and dead cells, Hoechst Hoechst 33258 pentahydrate (bis-benzamide) (Invitrogen) was added to cell suspensions after FASC wash and signals were determined on FACSymphony instrument (BD Biosciences, San Jose, CA, USA). Data were analyzed with FlowJo V10.10.0 (BD Biosciences).

Flow cytometry-based binding assay of BTCEs

TF-1, RPMI-8226 and U937 cell lines, human PBMCs or purified T cells (2-5x10^5) were washed in FACS wash buffer, re-suspended in 50 µL of BTCE solutions at indicated concentrations in FACS wash buffer and incubated on ice for 2 hours. For negative control, human PBMCs or T cells were pre-incubated with Purified NA/LE mouse anti-human CD3Ɛ (Clone UCHT1, BD) (10 µg/mL) at 4 °C for 30 minutes. Cells were washed twice and stained with Alexa Fluor 647 goat anti-human-$F(ab')_2$ fragment (Jackson ImmunoResearch Laboratories, PA, USA) for the detection of the BTCE at room temperature (RT) for 1 hour followed by two washes in FACS wash buffer and fixation in paraformaldehyde (1%) + CaCl2 (2.5 mM) solution. Median fluorescence intensity (MFI) ratios were calculated with the equation: MFI ratio = [MFI stained samples TCE-treated] / MFI stained samples TCE-untreated]. BTCE levels were determined by flow cytometry with a BD LSRII-Fortessa (BD Biosciences) and data were analyzed with FlowJo (LLC, CA, USA) software.

To determine the residual BTCE on human T cells, spleen samples of untreated and CD117xCD3 BTCE huCD34 NSG were taken. Frozen splenocytes were thawed and washed in FACS wash buffer. For CD117xCD3 BTCE positive binding control, splenocytes were re-suspended in 50 µL of CD117xCD3 BTCE solutions at 1.1 nM in FACS wash buffer and incubated on ice for 2 hours. Cells were washed twice and stained with Phycoerythrin (PE) goat anti-human-$F(ab')_2$ fragment

(Jackson ImmunoResearch Laboratories, PA, USA) at RT for 1 hour followed by two washes in FACS wash buffer. Samples were acquired by flow cytometry with a BD LSRII-Fortessa (BD Biosciences) and data were analyzed with FlowJo (LLC, CA, USA) software.

T cell-dependent cellular cytotoxicity (TDCC) assay

Target cells (cell lines or BM huCD34+ cells) were labeled with CFSE CellTrace™ fluorescent dye (ThermoFisher Scientific) according to the manufacturer's protocol. CellTrace labeled cells ($1x10^6$ cells/mL, 50 µL/well) were seeded in a 96-well round-bottom plate and BTCEs (50 µL, at indicated concentrations) were added to the wells and incubated for 30 minutes. Next, 100 µL human purified T cells at indicated effector-to-target (E:T) ratios in complete medium A or B (see above) was added to the cells and incubated at 37°C, 5% CO_2 for 6 or 24 hours (cell lines) or 48 hours (PDX and primary AML samples). After two washes, cells were stained with Annexin-V-PerCP-Cy5.5 or Annexin-V-APC. Cells were analyzed by flow cytometry with at least 10,000 total events within the target cell gate were collected. To measure the specific target cell lysis, CellTrace+ Annexin-V+ cells or CD117+ Annexin-V+ cells were gated. The percentage of specific lysis was then calculated using the following equation: Cytotoxicity (%) = [(% induced apoptosis - % spontaneous apoptosis) / (100 - % spontaneous apoptosis)] X 100, as previously published (2). PDX cells (see above) used in these assays were t(8;21) ([AML-PDX-CD117$^{Asp(816)-Val}$] while primary adult AML samples harbored a NPM1 mutation and in-frame bZIP mutated CEBPA (Sample 1), an NPM1 mutation (Sample 2), NPM1, IDH1, PTPN11 and FLT3-TKD mutations (Sample 3), and ASXL1, IDH1, EZH2, and STAG2 mutations (Sample 4).

Cross-activity test BTCE on murine T cells

Murine CD3+ cells were purified from spleens of C57BL6/J mice by negative selection with magnetic beads (EasySep, Stemcell Technologies). To measure the cross-activity of BTCEs, TF-1 target cells were labeled with FarRed CellTrace fluorescent dye (ThermoFisher Scientific), according to the manufacturer's protocol. BTCEs (10.1 nM) in complete medium A were added to each well. Next, CellTrace-labeled TF-1 target cells ($1x10^6$ cells/mL, 50 µL) were plated in complete medium A in a 96-well round-bottom plate and 100 µL purified murine T cells (E:T = 4:1) were added and plates were incubated at 37°C, 5% CO_2 for 24 hours.. To analyze the murine T cell-induced specific target cell lysis, the cells were harvested and stained with Annexin-V-BV421 and anti-mouse antibodies including mCD3-PerCp-eF710, mCD4-PE-CF594 and mCD8-BV711. The TF-1 target cells (CellTrace + Annexin-V+) were examined using flow cytometry and the same gate strategy used for the TDCC assay described above.

T cell activation assay

To measure the T cell activation, cells from TDCC assays were harvested after 24 or 48 hours. Cells were stained with anti-human antibodies including huCD3-BV421, huCD4-BV650, huCD8-BV786, huCD69-BV711, huCD25-SB600 and Annexin-V-APC. CFSE CellTrace-labeled target cells or CD3- cells were excluded from the analysis. Annexin-V- CD4+ and Annexin-V- CD8+ T cells were analyzed for CD25 and CD69 expression with FlowJo V10.10.0 (BD Biosciences).

T cell proliferation assay

To analyze T cell proliferation, $0.5x10^5$ TF-1 target cells were incubated with FarRed CellTrace-labeled human purified T cells (E:T = 10:1) in complete medium A (see above) containing indicated BTCEs at indicated concentrations at 37 °C for 5 days. Cells were harvested and stained with huCD33-PE, huCD4-BV650, huCD8-BV876 and Annexin-V-BV421. Data were acquired with BD LSRII-Fortessa (BD Biosciences). Proliferation of T cells (Annexin-V- CellTrace+) was analyzed with FlowJo VX (BD Biosciences).

Animal studies

We used different mouse models as indicated in the experimental sections below. Animals are randomly assigned to the different experimental groups. In all experiments we compare single animals from the CD117xCD3 reagent-treated groups with HELxCD3 control-treated groups. In all experiments performed a total of 4 to 5 mice per group were considered as sufficient for reaching statistical significance. All data from animals in the experiments were used for analysis, no exclusion criteria were used. Primary outcomes in experiments are HSC depletion and HSC chimerism. All animals were housed at the same location under same conditions to exclude confounder effects.

Mouse experiments at Erasmus MC were performed under project license nr. 2010607 provided by the animal welfare body (AWB) of the “Instantie voor Dierenwelzijn (IvD)”. All animal experiments were conducted in compliance with the Netherlands’ government laws of the “Centrale Commissie Dierproeven (CCD)”. All mice were housed in a certified barrier facility at Erasmus University Medical Center (Erasmus MC). Mouse experiments at the Institute of Molecular Genetics of the Czech Academy of Sciences were performed under project license nr.

AVCR 7450/2022 SOV II. AML-PDX-transplanted mice were maintained under specific pathogen free conditions in the animal facility of the Institute of Molecular Genetics of the CAS.

In vivo pharmacokinetics (pK) of CD117xCD3 BTCE

Female C57BL6/J mice (8-10 week old) (Charles River France) were injected intraperitoneally (IP) with 25 µg (1.0 mg/kg) of CD117xCD3 BTCE diluted in sterile saline (0.9%). Blood samples (20-50 µL) were collected from the tail vein into Microvette CB300 Capillary Blood Collection Tube with clotting activator (Sarstedt) at different time-points post-CD117xCD3 BTCE injection. After serum isolation by centrifugation at 10,000 x g at RT for 5 minutes, the serum samples were aliquot and stored at -20°C. The CD117xCD3 BTCE concentrations in serum were measured by enzyme-linked immunosorbent assay (ELISA) within for 4 weeks post-isolation. For ELISA assay, human recombinant CD117 protein isoform 2 (P10721-2), extracellular domain (Met 1-Thr 516) and with a poly histidine (His) tag at the C-terminus (Bio-Connect BV) (2 µg/mL in coating buffer [carbonate/bicarbonate buffer]) was used to coat the multi-array 96-well sector plates at 4 °C overnight. Plates were washed three times with 200 µL of PBST (PBS plus Tween-20 [0.05%]), and blocked with PBST BSA (5%) at RT for 2 hours. After thawing on ice, samples were centrifuged at 13,000 x g at 4°C for 5 minutes. Samples were diluted (1:100 - 1:2000) in dilution buffer (PBST BSA [1%]) and incubated at 4°C overnight. For BTCE detection and after washing with PBST, goat anti-Human IgG, 100 µL F(ab')$_2$ fragment specific and HRP conjugated antibody (Jackson ImmunoResearch Laboratories, PA, USA) (1:10,000 in blocking buffer) was added at RT for 1 hour on a shaker. After washing, 50 µL 3,3’,5,5’-tetramethylbenzidine solution (TMB) solution was added to the wells for at least 15 minutes. Next, 50 µL H_2SO_4, 2N (stopping solution) was added.

The signal was detected with a Versamax microplate reader (Molecular Devices) at 450/650 nm. The samples for the calibration curve with concentrations ranging from 20 to 0.039 µg/mL CD117 protein were prepared by spiking in pooled blank serum samples from BTCE-untreated mice.

BTCE treatment of humanized NSG mice

Female mice (16 to 24-weeks old) transplanted with human CD34+ hematopoietic stem cells (huCD34-NSG (#005557), were purchased from The Jackson Laboratory. PB samples were collected from tail vein one day before BTCE treatment. PB cells (huCD45+, huCD3+, huCD4+ and huCD8+) were stained with mCD45-AF700, huCD45-FITC, huCD3-PE-CF594, huCD4-BV650, huCD8-BV876 and Annexin-V-PerCP-5.5 and analyzed by flow cytometry. Mice containing >50% of human CD45+ cells in PB were used for experiments. Mice containing abnormal maturation of human T cells were excluded. BTCEs were diluted in sterile saline solution (0.9%) and 25 µg (1.0 mg/kg) were injected IP every 12 hours and in total 6 times. Sixteen hours, 5 and 7 days post-BTCE treatment, mice were sacrificed by cervical dislocation and BM cells from femur and tibia, spleens and peripheral blood cells were collected. HSPCs were stained with mCD45-AF700, human Lineage-BV510, huCD45-FITC, huCD34-APC, huCD117-BV421 and Annexin-V-PerCP-Cy5.5. Human lymphocytes were stained with mCD45-AF700, huCD45-FITC, huCD3-PE-CF594, huCD4-BV650, huCD8-BV786, huCD11b-APC, huCD11c-BV711, huCD13/huCD33-PE, huCD16-BV510, huCD14-PE-Cy7, huCD19-PerCP-Cy5.5, huCD56-PE-Cy5.5, Annexin-V-BV421 and cell subtypes were determined by flow cytometry. Data were acquired with the BD LSRII-Fortessa (BD Biosciences).

HSCT of allogeneic human PB CD34+ cells into BTCE-treated huCD34-NSG mice

On day 1 post-BTCE treatment (as described above), huCD34-NSG (HLA-A3+ HLA-A2-) mice were IP injected with 750 µg of anti-histamine Diphenhydramine hydrochloride (DPH) (Merck Chemicals BV) and intravenously (IV) injected with 500 µg of ACK2 (Ultra-LEAF, BioLegend Europe BV). DPH is included to counter the anaphylactic effects of mast cell triggering which ACK2 antibody, which is a known effect in mice of NOD genetic background (2). On day 5 post-BTCE treatment, huCD34-NSG (HLA-A3+/HLA-A2-) mice received 200 µg OKT3 (anti-human CD3 antibody, BioLegend Europe BV) by IP injection. On day 7 post-BTCE treatment, huCD34-NSG (HLA-A3+ HLA-A2-) mice were transplanted via tail vein injection with 200,000 PB-CD34+ (HLA-A3- HLA-A2+) cells. At 6 weeks post-transplantation, the percentage of human donor cells (HLA-A3- HLA-A2+) in PB and BM was monitored. Detection of chimerism by flow cytometry was performed with antibodies against human HLA-A3 and human HLA-A2, which were expressed on the recipient and donor cells, respectively. To determine the chimerism of HSCs, cells were stained with mCD45-AF700, huCD45-BV510, huCD34-APC, huCD117-BV421, huHLA-A2-PE, huHLA-A3-FITC and Annexin-V-PerCP-Cy5.5. To determine the chimerism on total human (CD45+) cells, samples were stained with mCD45-AF700, huCD45-BV510, huHLA-A2-PE, huHLA-A3-FITC, huCD3-PE-CF594, huCD20-BV605, huCD14-PE-Cy7, huCD56-BV786, Annexin-V-APC (Supplementary Figure 5). Donor cell chimerism was calculated as HLA-A2+ HLA-A3- ratio of total human cells (CD45+), human B cells (CD45+ CD20+), human T cells (CD45+ CD3+), human myeloid cells (CD45+ CD13+ 33+), human NK cells (CD45+ CD56+), human monocytes (CD45+ CD14+), human CD34+ HSPCs (CD45+ CD34+ CD117+). For the short tandem repeat (STR) analysis, DNA was isolated with the DNeasy Blood & Tissue Kit by QIAGEN according to manufacturer's

instructions, from BM samples at 6 weeks post-transplantation. Detection of chimerism by PCR was performed using AmpFLSTR™ Identifiler™ PCR Amplification Kit (ThermoFisher Scientific) according to the manufacturer's protocol. Data were obtained with a 3500 Series Genetic Analyzer (ThermoFisher Scientific) and analyzed with ChimeRMarker software (SoftGenetics).

BTCE treatment of AML PDX mice and HSCT

PDX-AML cells with normal karyotype and FLT3-ITD ($1x10^6$) were transplanted into sub-lethally irradiated (0.8 Gy) 7-9-week-old female NOD.Cg-Prkdcscid Il2rgtm1Wjl Tg(CMV IL3, CSF2, KITLG) 1Eav/MloySzJ (NSG-SGM3) mice (The Jackson Laboratory, Bar Harbour, ME, USA) by tail vein injection and expanded *in vivo* for 3 weeks. Human T cells were purified from frozen PBMCs as described above and activated *in vitro* for 72 hours using the T Cell TransAct kit (Miltenyi Biotec), according to the manufacturer's instructions. Three weeks post-PDX-AML transplantation, $10x10^6$ pre-activated human T cells were injected IP, followed by BTCE treatments after 12 hours for 3 days (25 µg per injection, 2 injections per day). Next, PDX-AML mice were injected IP with 750 µg DPH and IV with 500 µg ACK2 at 36 hours post-BTCE treatment and IP with 200 µg of OKT3 at 60 hours post-BTCE treatment. Four days post-BTCE treatment, mice were transplanted with 150,000 human mismatched PB-CD34+ (HLA-A3- HLA-B7+) cells by tail vein injection. Mice were sacrificed and BM cells were isolated from femur and tibia. The percentage of healthy donor cells in the BM of mice was determined by flow cytometry using anti-human antibodies against HLA-A3 and HLA-B7, which are expressed on the recipient and donor cells, respectively. To determine the chimerism, BM cells were stained with mCD45-AF700, huCD45-BV510, huCD34-

PE-Cy7, huCD117-BV421, huHLA-A3-FITC, huHLA-B7-APC, Annexin-V-PerCP-Cy5.5 and analyzed with the BD LSRII-Fortessa (BD Biosciences) 6 weeks post-transplantation.

Secondary transplantation of PDX-AML BM cells post-BTCE treatment

NSG-SGM3 mice were transplanted with PDX-AML cells and treated with BTCE as described above. Mice were sacrificed 36 hours post-BTCE treatment and the presence of PDX AML cells and human T cells was assessed by flow cytometry with mCD45-AF700, huCD45-FITC, huCD34-APC, huCD117-BV421, huCD33-PE, huCD3-PE-CF594, huCD4-BV650, huCD8-BV786, Annexin-V-PerCP-Cy5.5. Human T cells in the BM samples were depleted with the EasySep™ Human CD3 Positive Selection Kit II (StemCell Technologies). Next, $0.2x10^6$ human T cell-depleted BM cells were transplanted into sub-lethally irradiated (0.8 Gy) 4-6-week-old female NSG-SGM3 mice. Blood samples (20-50 µL) were monthly taken from the cheek vein and the percentage of primary PDX-AML cells in PB was monitored by flow cytometry with mCD45.1-FITC, huCD45-APC-Cy7 and Hoechst 33258. Mice were sacrificed 6 months post-transplantation and the presence of PDX-AML cells in the BM, spleen and PB of transplanted mice was determined by flow cytometry as described above. Data were acquired with the FACSymphony instrument (BD Biosciences, San Jose, CA, USA).

Cytokine analysis

To examine the human cytokine production in response to BTCE treatment *in vitro*, supernatants were collected from the TDCC assay and centrifuged to remove residual debris or intact cell. Serum was isolated at the indicated time points. All samples were stored at -20°C for a maximum

of one month. After thawing samples on ice, they were centrifuged at 13,000 g for 5 minutes at 4°C. Two-fold dilutions were prepared in low-protein-binding plates (Corning™ 96-Well Nonbinding Surface [NBS™] Microplates). Samples were diluted in wash buffer at 1:1 in wash buffer according to the manufacturer's instructions for supernatant. Diluted samples were loaded into ELLA Simple Plex cartridges and analyzed with the ELLA Simple Plex system according to the manufacturer's instructions (Ella automated Immunoassay system, Biotechne).

High-dimensional flow cytometry

BM samples of huCD34-NSG mice 5 days post-treatment were stained with a 41-color antibody panel (**Supplementary Table 2**) and data was collected using a 5 laser Aurora spectral flow cytometer (Cytek Biosciences, CA). All samples were stained as described previously (3) with the adaptation of including Annexin-V-AF350 and mCD45-AF700 to exclude dead cells and mouse cells from the analysis, respectively. Cleaning of flow data was performed with SpectroFlo (version 2.2.0). The unsupervised, and statistical inference portions of the flow cytometry analysis were performed using OMIQ data analysis software (www.omiq.ai). We used the unsupervised analysis methods based on surface markers without any 2D gating we previously employed. The workflow included running flowCut to check for changes in channels over acquisition time, UMAP for dimensionality reduction, flowSOM for clustering, and edgeR for statistical inference. For the statistical comparisons of abundance, the Flow Cytometry Standard (FCS) files were subsampled to ensure the same number of events were included per group.

Statistics

All statistical analyses were performed with GraphPad Prism 9 (GraphPad, La Jolla, CA, USA). The two-tailed Student's t-test or a one-way ANOVA with Dunnett's multiple comparisons test was used for statistical analysis. Statistics are indicated in the figure legends. Four-parameter variable slope nonlinear regression was used for dose-response curve fitting and PK analysis.

References

1. Blumberg RS, Ley S, Sancho J, Lonberg N, Lacy E, McDermott F, et al. Structure of the T-cell antigen receptor: evidence for two CD3 epsilon subunits in the T-cell receptor-CD3 complex. Proc Natl Acad Sci U S A. 1990;87(18):7220-4.
2. Mueller YM, De Rosa SC, Hutton JA, Witek J, Roederer M, Altman JD, Katsikis PD. Increased CD95/Fas-induced apoptosis of HIV-specific CD8(+) T cells. Immunity. 2001;15(6):871-82
3. Louvet C, Szot GL, Lang J, Lee MR, Martinier N, Bollag G, et al. Tyrosine kinase inhibitors reverse type 1 diabetes in nonobese diabetic mice. Proc Natl Acad Sci U S A. 2008;105(48):18895-900.
4. Park LM, Lannigan J, Jaimes MC. OMIP-069: Forty-Color Full Spectrum Flow Cytometry Panel for Deep Immunophenotyping of Major Cell Subsets in Human Peripheral Blood. Cytometry A. 2020;97(10):1044-51.
